# Biomarkers associated with functional improvement after stroke rehabilitation: a systematic review and meta-analysis of randomized controlled trials

**DOI:** 10.3389/fneur.2023.1241521

**Published:** 2023-09-05

**Authors:** Gengbin Chen, Manfeng Wu, Jialin Chen, Cailing Zhang, Quan Liu, Yinchun Zhao, Guangqing Xu, Yue Lan

**Affiliations:** ^1^Department of Rehabilitation Medicine, Guangzhou First People’s Hospital, School of Medicine, South China University of Technology, Guangzhou, China; ^2^Postgraduate Research Institute, Guangzhou Sport University, Guangzhou, China; ^3^Department of Rehabilitation Medicine, Guangdong Provincial People’s Hospital, Guangdong Academy of Medical Sciences, Guangzhou, China; ^4^Guangzhou Key Laboratory of Aging Frailty and Neurorehabilitation, Guangzhou, China

**Keywords:** meta-analysis, rehabilitation, biomarker, blood, function, stroke

## Abstract

**Objective:**

This study aims to identify blood and cerebrospinal fluid biomarkers that are correlated to the functional improvement of stroke patients after rehabilitation therapy, and provide ideas for the treatment and evaluation of stroke patients.

**Methods:**

The PubMed, Web of Science, and Embase databases were searched for articles published in the English language, from inception to December 8, 2022.

**Results:**

A total of 9,810 independent records generated 50 high-quality randomized controlled trials on 119 biomarkers. Among these records, 37 articles were included for the meta-analysis (with a total of 2,567 stroke patients), and 101 peripheral blood and cerebrospinal fluid biomarkers were included for the qualitative analysis. The quantitative analysis results revealed a moderate quality evidence that stroke rehabilitation significantly increased the level of brain-derived neurotrophic factor (BDNF) in serum. Furthermore, the low-quality evidence revealed that stroke rehabilitation significantly increased the concentration of serum noradrenaline (NE), peripheral blood superoxide dismutase (SOD), peripheral blood albumin (ALB), peripheral blood hemoglobin (HB), and peripheral blood catalase (CAT), but significantly decreased the concentration of serum endothelin (ET) and glutamate. In addition, the changes in concentration of these biomarkers were associated with significant improvements in post-stroke function. The serum BNDF suggests that this can be used as a biomarker for non-invasive brain stimulation (NIBS) therapy, and to predict the improvement of stroke patients.

**Conclusion:**

The concentration of serum BNDF, NE, ET and glutamate, and peripheral blood SOD, ALB, HB and CAT may suggest the function improvement of stroke patients.

## Introduction

Stroke is the leading cause of death in China, and the second leading cause of death worldwide (1, 2). Surviving a stroke can lead to a series of sequelae, such as post-stroke motor deficits, sensory deficits, cognitive deficits, and other dysfunctions, increasing the global medical burden (3–6). Rehabilitation plays an essential role in the functional recovery of post-stroke patients (7). To date, a number of general scales and instruments (National Institute of Health Stroke Scale, Barthel index, etc.) have been introduced worldwide to evaluate the recovery of extrinsic function (motor function, cognitive function, speech function, etc.) of stroke patients (8). However, relatively few studies (9, 10) have evaluated the intrinsic physiological mechanism of recovery (improvement of neural repair and protection of brain tissues) of stroke patients, in order to predict possible treatment targets through changes in blood and cerebrospinal fluid components, before and after intervention. Furthermore, since relevant evidences have not been summarized by any published literature, it remains difficult to determine whether the changes in biomarkers can predict the functional improvement or deterioration of stroke patients.

In order to provide the possibility of developing biomarkers for improved function in stroke rehabilitation, and put forward specific therapeutic targets, the present study conducted a meta-analysis and systematic evaluation of evidence-based treatments, and documented the presence or absence of biomarkers that show evidence of replication. The present study aims to identify biomarkers associated with functional improvement after the rehabilitation of stroke patients.

## Methods

### Data sources and search strategy

The present meta-analysis was registered in the INPLASY International Platform for Registered Systematic Reviews and Meta-Analyses Program (Registration number: INPLASY202320058), and performed according to the Preferred Reporting Items for Systematic Reviews and Meta-Analyses (PRISMA) standards.

A comprehensive literature search was conducted on three online databases (PubMed, Web of Science, and Embase) to identify relevant articles published in the English language, from inception to December 8, 2022. The search terms used for these databases were modified, and are listed in Supplementary Table S1. In addition, the reference list of the included articles was manually checked to identify relevant studies that did not appear in the literature search.

### Eligibility criteria

The inclusion criteria for the present study were, as follows: (1) randomized controlled trials (RCTs) that reported the therapeutic effect of rehabilitation therapy on adult stroke patients; (2) the intervention group received rehabilitation therapy alone or combined with other therapies, while the control group received a sham rehabilitation therapy or no rehabilitation treatment; and (3) the outcome included the concentration of biomarkers in peripheral blood (serum, plasma, etc.) or cerebrospinal fluid.

The exclusion criteria were, as follows: (1) studies that failed to meet the inclusion criteria; (2) study designs other than RCTs, such as observational studies; and (3) studies published in languages other than the English language.

At least three studies were required to quantitatively combine these for the meta-analysis. When less than three datasets were reported for a specific biomarker in a given biological fluid, the findings were qualitatively summarized.

### Data extraction

Three independent reviewers (CGB, WMF, and ZCL) assessed the eligibility of each study, and performed the data extraction and quality assessment of qualified studies. If there were any discrepancies, these were resolved by consensus.

The information obtained from each study included the first author’s name, publication year, number of participants, patient characteristics (age, gender, type of stroke, and average time to stroke), rehabilitation prescription, comprehensive training/practice, biomarker measurements (biological fluid and quantification method), and functional measurement.

For each trial, the mean differences and standard deviations of the outcomes at pre- and post-intervention were extracted for each group (rehabilitation and control groups). For studies without numerical data, the GetData Graph Digitizer 2.25 was used to extract the data from the graphs, or the corresponding author was contacted to request for any missing data.

### Quality assessment

The methodological quality of the included studies was assessed by three independent raters using the Physiotherapy Evidence Database (PEDro) scale (11, 12). The scale consisted of 11 elements. The first element was the measure of external validity, but this was not taken into account when the overall results were calculated. Each of the 10 quality criteria was marked as 1 (pass) or 0 (fail). Individual item scores were added to determine each study’s total score. The maximum total score for each study was 10/10. In addition, the Grading of Recommendation Assessment, Development and Evaluation (GRADE) method was used to determine the quality of evidence provided by the RCTs (13). This included five standards: risk of bias, inconsistent results, imprecision of results, indirectness of evidence, and publication bias. The quality of each piece of evidence was categorized as high, moderate, low, or very low.

### Data synthesis and analysis

Based on the results for eligible studies, a further functional meta-analysis was conducted for studies with significant differences in biomarkers, in order to determine the therapeutic effect of the rehabilitation. Since the functional measurement values of these studies were not completely consistent, the data were extracted according to the research objectives of each study. If nearly 10 observations were compared, three subgroup analyses were performed to determine the factors that influenced the changes in biomarker concentrations induced by the stroke rehabilitation: (1) stroke stage, (2) rehabilitation method, and (3) treatment sessions (non-invasive brain stimulation, NIBS).

All statistical analyses were carried out using the Stata MP 14.0 software. The standardized mean differences (SMDs) of the change scores (endpoints minus baseline scores) and the corresponding 95% confidence intervals (CIs) were used to summarize the effect. The heterogeneity was evaluated using the *I^2^* statistic and Cochrane’s Q-test. When low heterogeneity was observed (*I^2^* < 50%, *p* > 0.05), the fixed-effects model was used. Otherwise, the random effects model was used. A *p*-value of 0.05 was considered statistically significant. A sensitivity analysis was performed to assess the stability of the systematic studies.

## Results

### Characteristics of the RCTs

**Figure 1:**
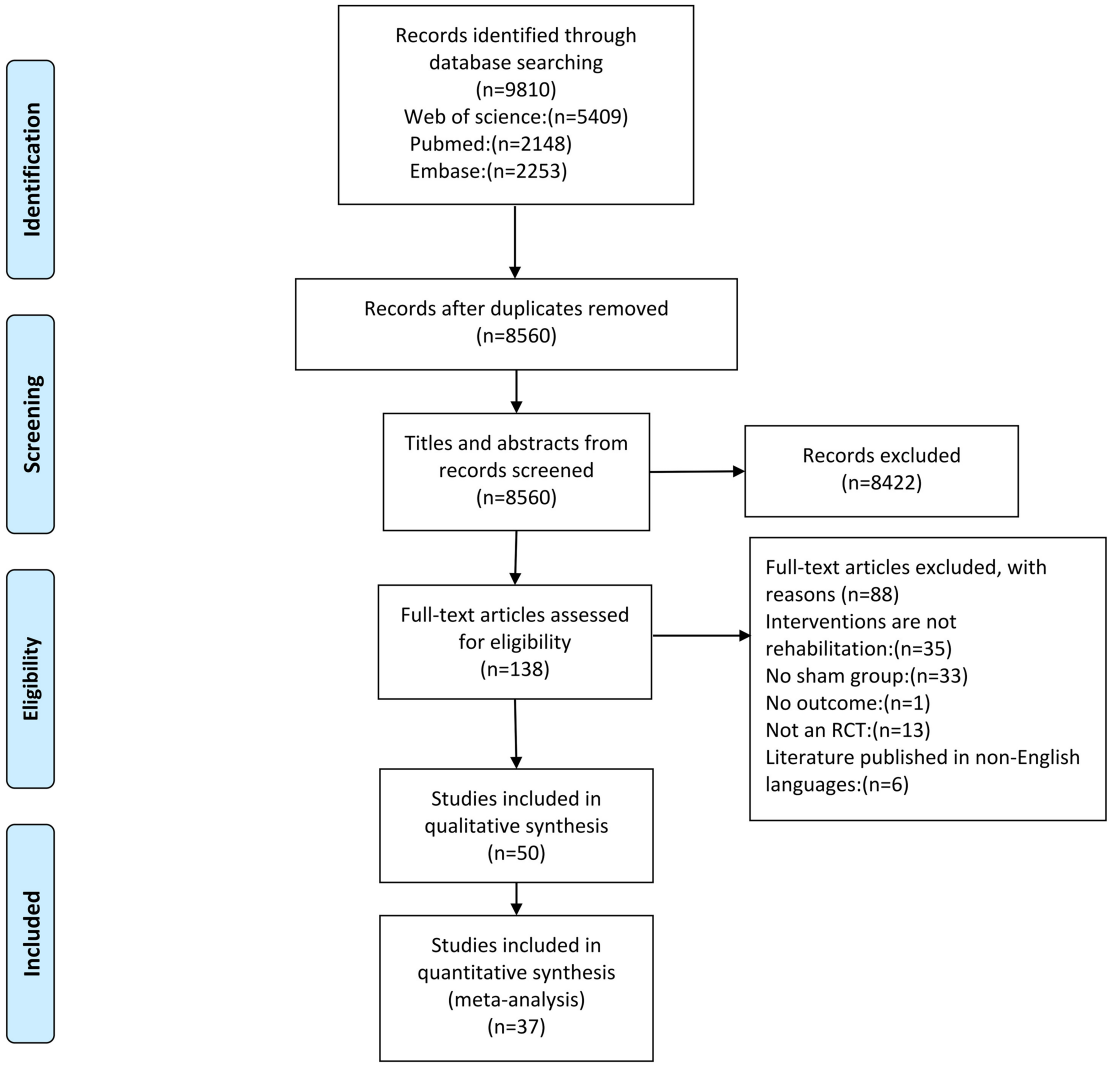
The PRISMA flowchart for the selection and inclusion of studies.

The database search identified a total of 9,810 articles (Figure 1). Among the 50 studies that met the inclusion criteria, 36 studies were entered into the qualitative review form (Supplementary Table S2), and 37 high-quality RCTs were included for the meta-analysis (with a total of 2,567 stroke patients). The details of the included studies are presented in Supplementary Table S2. Sub-studies were identified in one study, which included two experimental groups (10). The serum meta-analysis included six markers, the plasma meta-analysis included two markers, and the peripheral blood meta-analysis included 10 markers (Table 1). For the qualitative synthesis, 101 kinds of markers in plasma, serum, peripheral blood, and cerebrospinal fluid were included (Supplementary Table S3).

**Table 1:** Summary of meta-analyses.

	Studies (*n*)	SMD (95% CI)	*P*	*I^2^* (%)	*P* (heterogeneity)
**Serum**					
**BDNF**	10	1.57 (0.70, 2.44)	0	93.1	0
(function)	9	1.45 (0.94, 1.97)	0	80.4	0
Stage of stroke					
Acute	1	0.21 (−0.18, 0.61)	0.29		
Subacute	9	1.76 (0.77, 2.76)	0.001	92.8	0
Rehabilitation method					
NIBS	8	1.94 (0.82, 3.06)	0.001	93.5	0
no-NIBS	2	0.30 (−0.05, 0.65)	0.094	0	0.350
Treatment sessions (NIBS)					
5 sessions	1	0.14 (−0.70, 0.98)	0.746		
10 sessions	2	1.14 (−1.12, 3.41)	0.322	90.6	0.001
18–20 sessions	5	2.23 (0.82, 3.64)	0.002	93.3	0
30+ sessions	2	4.75 (1.30, 8.20)	0.007	87.2	0.005
**TNF-α**	3	−2.19 (−4.13, −0.24)	0.027	98.1	0
(function)	3	0.83 (−0.02, 1.69)	0.057	93.8	0
**ET**	3	−2.29 (−4.48, −0.10)	0.041	98.3	0
(function)	3	0.42 (0.08, 0.75)	0.014	56.4	0.101
**NE**	3	0.94 (0.33, 1.54)	0.002	80.3	0.006
(function)	3	1.70 (1.09, 2.31)	0	76.7	0.014
**Glutamate**	3	−0.92 (−1.34, −0.51)	0	57.9	0.093
(function)	2	1.49 (0.06, 2.92)	0.041	91.9	0
**5-HT**	3	0.76 (−0.39, 1.91)	0.194	94.5	0
**Plasma**					
**VEGF**	3	1.84 (−0.16, 3.84)	0.071	95.6	0
**BDNF**	4	0.96 (−0.68, 2.59)	0.250	95.7	0
**Blood**					
**SOD**	4	4.17 (1.52, 6.82)	0.002	97.2	0
(function)	3	1.05 (0.71, 1.40)	0	0	0.555
**ALB**	3	1.45 (0.31, 2.58)	0.013	93.9	0
(function)	3	0.65 (0.41, 0.89)	0	0	0.617
**HB**	5	1.62 (0.62, 2.62)	0.001	93.8	0
(function)	5	0.63 (0.42, 0.84)	0	0	0.615
**CAT**	3	11.87 (5.98, 17.76)	0	98.5	0
(function)	2	0.97 (0.49, 1.44)	0	0	0.344
**CGRP**	3	0.10 (−0.94, 1.15)	0.844	93.4	0
**FBG**	3	−0.16 (−0.40, 0.08)	0.194	0	0.934
**TC**	6	−0.13 (−0.35, 0.08)	0.226	19.2	0.288
**TG**	3	−0.18 (−0.41, 0.05)	0.124	0	0.755
**LDL**	4	−0.18 (−0.39, 0.02)	0.083	0	0.915
**HDL**	6	0.10 (−0.09, 0.28)	0.308	0	0.831

BDNF, brain-derived neurotrophic factor; TNF-α, tumor necrosis factor-α; ET, endothelin; NE, noradrenaline; 5-HT, 5-hydroxytryptamine; VEGF, vascular endothelial growth factor; SOD, superoxide dismutase; ALB, albumin; HB, hemoglobin; CAT, catalase; CGRP, calcitonin-gene-related peptide; FBG, fasting blood glucose; TC, total cholesterol; HDL, high density lipoprotein; LDL, low density lipoprotein; TG, triglyceride; NIBS, non-invasive brain stimulation.

According to the PEDro score, the quality scores of the studies ranged within 6–10, with an average quality score of 7.14 ± 1.18 (mean ± standard deviation), indicating that the methodological quality was relatively high (Table 2). The quality of the evidence evaluated using the GRADE method is presented in Supplementary Table S4. The sensitivity analysis results revealed that these had no significant influence on the meta-analysis results (Supplementary Figure S1).

**Table 2:** Assessment of risk of bias for the included studies.

References	Criteria											Total
	1	2	3	4	5	6	7	8	9	10	11	
Bai et al. (14)	Y	1	0	1	1	0	0	1	1	1	1	7
Bai et al. (10)	Y	1	0	1	1	0	0	1	1	1	1	7
Bintang et al. (15)	Y	1	0	1	1	0	1	1	1	1	1	8
Carr et al. (16)	Y	1	0	1	0	0	0	1	1	1	1	6
Cichon et al. (9)	Y	1	0	1	1	0	0	1	1	1	1	7
Cichon et al. (17)	Y	1	0	1	1	0	0	1	1	1	1	7
Faulkner et al. (18)	Y	1	1	1	0	0	1	1	1	1	1	8
Gambassi et al. (19)	Y	1	0	1	0	0	0	1	1	1	1	6
Gjellesvik et al. (20)	Y	1	0	1	1	1	1	1	1	1	1	9
He et al. (21)	Y	1	0	1	0	0	0	1	1	1	1	6
Hsu et al. (22)	Y	1	0	1	1	1	1	1	1	1	1	9
Huang et al. (23)	Y	1	0	1	0	0	0	1	1	1	1	6
Krawcyk et al. (24)	Y	1	1	1	1	1	1	1	1	1	1	10
Lee et al. (25)	Y	1	0	1	1	0	1	1	1	1	1	8
Lennon et al. (26)	Y	1	1	1	0	0	1	1	1	1	1	8
Liang et al. (27)	Y	1	0	1	0	0	1	1	1	1	1	7
Liu et al. (28)	Y	1	0	1	0	0	0	1	1	1	1	6
Lu et al. (29)	Y	1	0	1	1	1	0	1	1	1	1	8
MacKay-Lyons et al. (30)	Y	1	1	1	0	0	1	1	1	1	1	8
Mao et al. (31)	Y	1	0	1	0	0	1	1	1	1	1	7
Qin et al. (32)	Y	1	0	1	1	0	1	1	1	1	1	8
Tang et al. (33)	Y	1	0	1	0	0	0	1	1	1	1	6
Utomo et al. (34)	Y	1	0	1	0	0	0	1	1	1	1	6
Vahlberg et al. (35)	Y	1	0	1	0	0	0	1	1	1	1	6
Wang et al. (36)	Y	1	0	1	0	0	0	1	1	1	1	6
Wang et al. (37)	Y	1	0	1	0	0	0	1	1	1	1	6
Wang et al. (38)	Y	1	0	1	0	0	0	1	1	1	1	6
Wang et al. (39)	Y	1	0	1	0	0	0	1	1	1	1	6
Wang et al. (40)	Y	1	0	1	1	1	0	1	1	1	1	8
Wang et al. (41)	Y	1	0	1	1	0	1	1	1	1	1	8
Wang et al. (42)	Y	1	0	1	0	0	0	1	1	1	1	6
Xiong et al. (43)	Y	1	0	1	1	0	1	1	1	1	1	8
Zhang et al. (44)	Y	1	0	1	0	0	0	1	1	1	1	6
Zhang et al. (45)	Y	1	0	1	0	0	0	1	1	1	1	6
Zhang et al. (46)	Y	1	0	1	0	0	0	1	1	1	1	6
Zhao et al. (47)	Y	1	1	1	1	1	0	1	1	1	1	9
Zhao et al. (48)	Y	1	1	1	1	0	1	1	1	1	1	9

Criteria numbers: 1, eligibility criteria; 2, random allocation; 3, concealed allocation; 4, similar groups at baseline; 5, blinding subjects; 6, blinding therapists; 7, blinding assessors; 8, outcome obtained in more than 85% of the subjects; 9, intention-to-treat analysis; 10, between-group statistical comparisons; 11, point estimates and measures of variability.

### Biomarkers in serum

**Figure 2:**
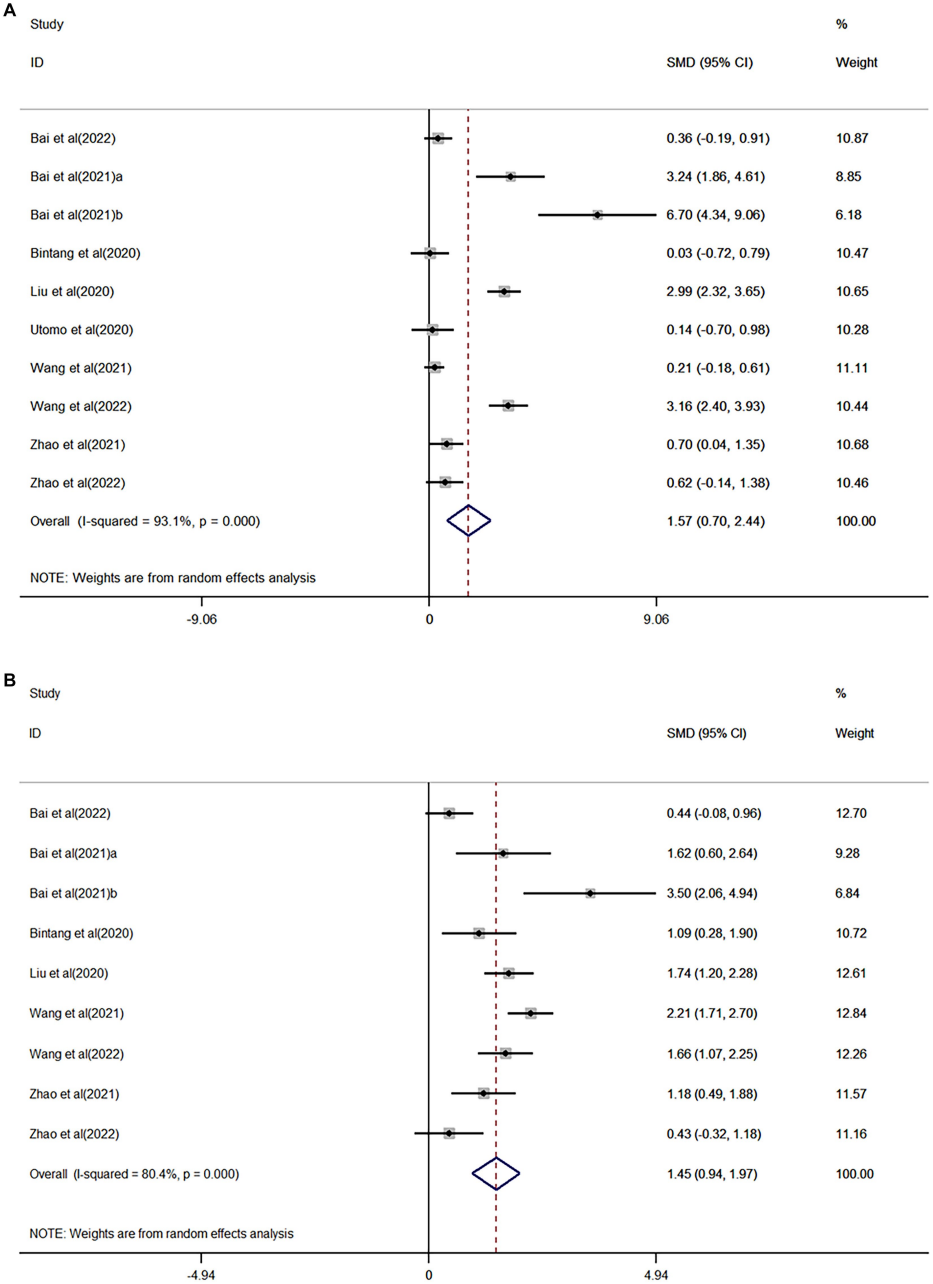
**(A)** Forest plot for the effect of rehabilitation treatment on serum brain-derived neurotrophic factor (BDNF) in stroke patients. **(B)** Forest plot for the effect of rehabilitation therapy on functional recovery in stroke patients in the serum BDNF study.

For the effect of the rehabilitation on serum biomarkers in stroke patients, the meta-analysis revealed a moderate evidence, when compared to the control group. Furthermore, the concentration of brain-derived neurotrophic factor (BDNF) significantly increased in the treatment group, and this was significantly correlated to the functional improvement after stroke (10, 14, 15, 28, 34, 36, 41, 47, 48) (Figure 2; Table 1). Compared to the control group, the low-quality evidence in the treatment group revealed that the concentration of serum noradrenaline (NE) increased (27, 28, 36), while the concentrations of serum endothelin (ET) (39, 44, 46) and glutamate (36, 40, 42) decreased, and these were significantly associated with the functional improvement after stroke (Supplementary Figures S2–S4; Table 1).

Compared to the control group, the concentration of serum tumor necrosis factor-α (TNF-α) (38, 39, 45) significantly decreased in the treatment group, but this had no significant correlation with the improvement in post-stroke function (Supplementary Figures S5; Table 1). The serum concentration of 5-hydroxytryptamine (5-HT) in the treatment group did not significantly change (Supplementary Figure S6; Table 1) (27, 28, 36).

For the effect of rehabilitation therapy on the serum BDNF concentration in stroke patients, the sub-group analysis based on the stroke period revealed that there was a significant correlation with the concentration changes in the subacute stage of stroke, but there was no correlation in the acute stage (36). The subgroup analysis based on the rehabilitation therapy revealed that there was a significant correlation with non-invasive brain stimulation (NIBS) therapy, but there was no correlation with no-NIBS (36, 48) (Supplementary Figures S7A,B; Table 1). According to the treatment sessions for NIBS (five sessions vs. 10 sessions vs. 18–20 sessions vs. 30+ sessions), it was found that the serum BDNF concentration was significantly correlated with 18–20 sessions (10, 14, 28, 47) and 30+ sessions (10, 41), but this was not associated with five sessions (34) and 10 sessions (10, 15) (Supplementary Figures S7C; Table 1).

In the qualitative synthesis (Supplementary Table S3), the serum concentrations for nerve growth factor (NGF), nitric oxide, interleukin (IL)-1, IL-4, triiodothyronine (T3), free triiodothyronine (FT3), thyroid stimulating hormone (TSH), total protein, γ-aminobutyric acid (GABA), and vascular endothelial growth factor (VEGF) were significantly higher in the treatment group, when compared to the control group. However, the serum concentrations for matrix metalloproteinase (MMP-9), TNF, IL-6, intercellular adhesion molecule (ICAM)-1, soluble intercellular adhesion molecule (sICAM), soluble vascular cell adhesion molecule (sVCAM), ET-1, substance P, soluble E-selectin, soluble protein-100B, s-100, Toll-like receptor 4 (TLR4), nuclear factor kappa-B (NF-κB), malondialdehyde (MDA), NT-proBNP, corticotropin releasing factor (CRF), myelin basic protein (MBP), neuron specific enolase (NSE), reactive oxygen species (ROS), and lipid hydrogen peroxide (LHP) significantly decreased. The concentrations for serum insulin-like growth factor-1, total serum thyroxine (T4), free thyroxine (FT4), bone-specific alkaline phosphatase (BAP), and C-telopeptide of type I collagen cross-links (CTx) did not significantly change.

### Biomarkers in plasma

For the effect of rehabilitation on plasma biomarkers in stroke patients, the meta-analysis revealed that the plasma concentrations for BDNF (9, 23, 29, 43) and VEGF (9, 24, 25) did not significantly increase in the treatment group, when compared to the control group (Supplementary Figures S8, S9; Table 1).

In the qualitative synthesis (Supplementary Table S3), the plasma concentrations for NGF, thiol, IL-1β, IL-1β mRNA, IL-2, IL-4, vascular cell adhesion molecule1 (VCAM-1), and endothelial progenitor cells (EPCs) were significantly higher in the treatment group, when compared to the control group. Furthermore, the plasma concentrations for carbonyl, thiobarbituric acid reactive substances (TBARS), ICAM-1, and insulin significantly decreased. However, the plasma concentrations for stromal cell-derived factor 1α (SDF-1α), total antioxidant status (TAS), NADPH oxidase, nitrite, nitrite peroxide (H2O2), TNF, IL-10, interferon γ (IFN-γ), transforming growth factor-β (TGF-β), P-selectin, E-selectin, tissue plasminogen activator (tPA), plasminogen activator inhibitor-1 (PAI-1), von-Willebrand factor (vWF), Copeptin, and TNF-α did not significantly differ.

### Biomarkers in peripheral blood

For the influence of rehabilitation therapy on biomarkers in peripheral blood in stroke patients, the meta-analysis revealed a low-quality evidence that the concentrations of superoxide dismutase (SOD) (17, 19, 27, 42), albumin (ALB) (31, 32, 37), hemoglobin (HB) (20, 22, 31, 37), and catalase (CAT) (17, 19, 42) significantly increased in peripheral blood in the treated group, and that this was significantly associated with improvement in function after stroke (Supplementary Figures S10–S13; Table 1).

The concentrations of other biomarkers in peripheral blood in the treatment group did not exhibit significant changes, which included calcitonin-gene-related peptide (CGRP) (21, 33, 46), blood glucose (fasting) (16, 18, 30), total cholesterol (TC) (16, 18, 20, 24, 26, 35), triglyceride (TG) (20, 24, 30), low-density lipoprotein (LDL) (20, 24, 30, 35), and high-density lipoprotein (HDL) (16, 18, 20, 24, 30, 35) (Supplementary Figures S14–S19; Table 1).

In the qualitative synthesis (Supplementary Table S3), the concentrations in peripheral blood for lactate, EPCs, prealbumin, lgA, lgM, and lgG significantly increased, while the concentrations in peripheral blood for hematocrit, monocyte-platelet aggregates (MPA), white blood cells, and C-reactive protein significantly decreased in the treated group. There were no significant changes in concentrations in peripheral blood for erythrocytes, HbA1c, C-peptide, NOS2mRNA, leukocytes, platelets, lipoprotein-associated phospholipase A2 (Lp-PLA2), asymmetric dimethylarginine (ADMA), Mono1, Mono2, Mono3, MPA3, and thrombin.

### Biomarkers in cerebrospinal fluid

The qualitative synthesis results (Supplementary Table S3) revealed that there were no significant differences in concentrations of somatostatin (SS) in the cerebrospinal fluid in the treated group and control group (49).

## Discussion

**Figure 3:**
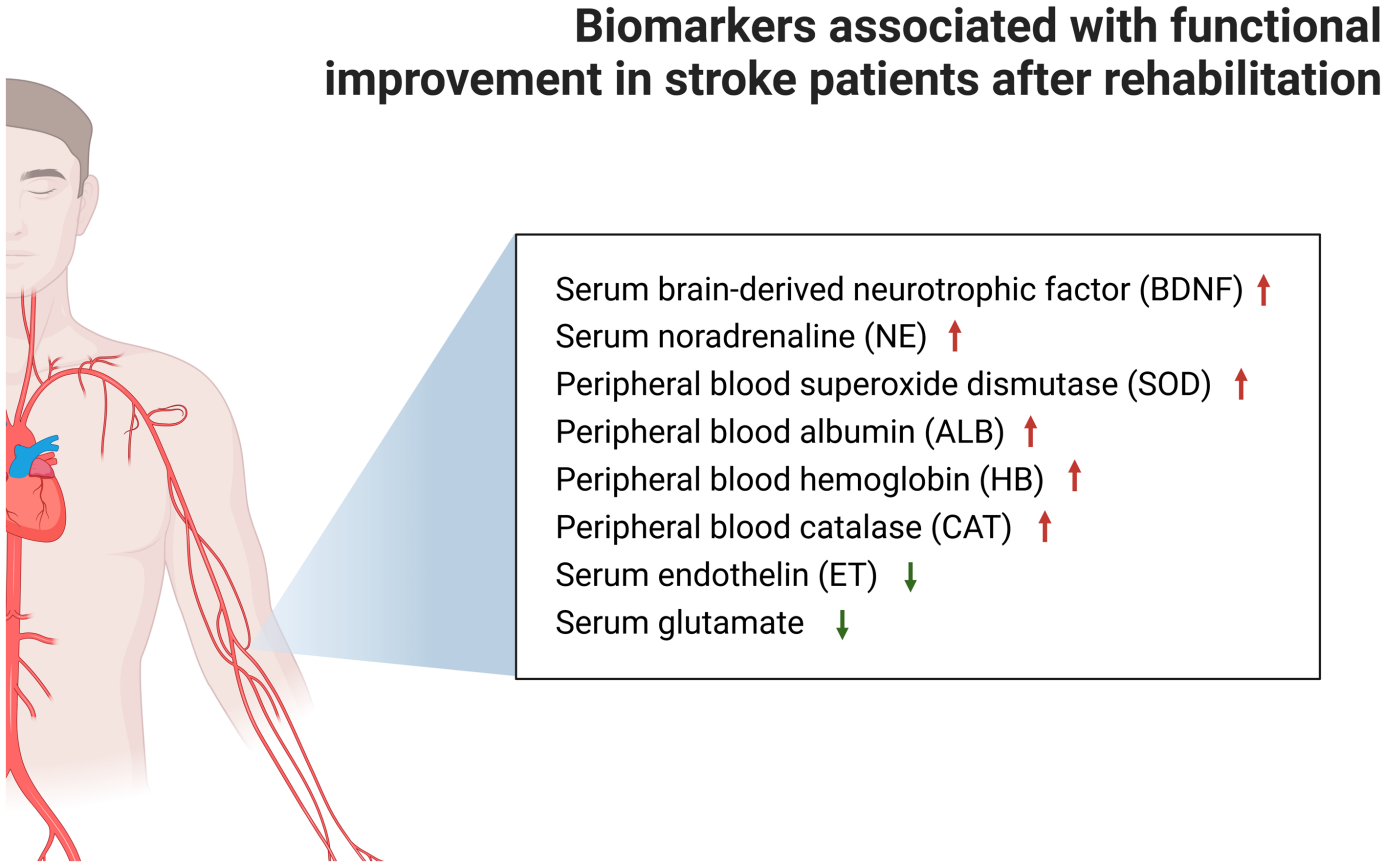
Markers associated with significant functional improvement after stroke rehabilitation are shown, while markers that could not be analyzed by the meta-analysis due to insufficient data were placed in Supplementary Table S2.

The rehabilitation prescriptions in the present study were mainly exercise training or NIBS. The meta-analysis of studies on biomarker changes after stroke rehabilitation revealed that rehabilitation can significantly increase the concentration of serum BDNF and NE, and peripheral blood SOD, ALB and HB, and CAT, and decrease the biomarkers of serum ET, glutamate, and TNF-α. In addition to serum TNF-α, the concentration changes of other biomarkers were significantly associated with functional improvement after stroke (Figure 3). The present study preliminarily deduced that serum BDNF, NE, ET and glutamate, and peripheral blood SOD, ALB, HB, and CAT can be used as indicators of functional recovery in stroke patients. Furthermore, the results revealed that some biomarkers did not exhibit flagrant concentration changes after rehabilitation therapy, such as plasma BDNF and VEGF, serum 5-HT, peripheral blood CGRP, glucose (fasting), TC, TG, LDL, and HDL.

Previous studies (50, 51) have repeatedly proven that low serum BDNF levels are significantly correlated to poor functional outcomes and high mortality, and that elevated BDNF levels after stroke are correlated to improvement of functional recovery. This is consistent with the present findings, in which the meta-analysis of the pooled data revealed that rehabilitation induced a significant increase in serum BDNF levels, and that this change was associated with functional improvement. The sub-group analysis for stroke period and treatment modality revealed that serum BDNF is closely correlated with the factors of subacute stroke and NIBS treatment, but not acute stroke and non-NIBS treatment. Due to the small number of studies on acute stroke and non-NIBS (there are only 1 and 2 studies, respectively), these results should be interpreted with caution, but there is no doubt that serum BDNF is significantly correlated with subacute stroke and NIBS treatment. The study (52) carried out by Niimi et al. confirmed that the combined rehabilitation of low-frequency rTMS appears to be able to improve the motor function of the affected limbs by activating BDNF. This indicates that serum BDNF can be used as a biomarker for NIBS treatment, and that this can be used to predict the improvement of stroke function. The subgroup analysis based on NIBS treatment times revealed that 18–20 or 30+ treatments had a significant effect, while 5 and 10 treatments did not have a significant effect. These results also revealed that the serum BDNF concentration increased with the increase in number of rehabilitation sessions.

For the other serum biomarkers that had significant effects after rehabilitation, it was found that the serum NE concentration significantly increased, while the serum ET and glutamate concentrations significantly decreased after the rehabilitation intervention, and this was correlated to the significant improvement in post-stroke function. This discovery reinforces the evidence obtained from previous studies conducted on animals and humans. Furthermore, previous studies (53–55) have revealed that NE can improve the motor network connectivity in stroke patients, thereby facilitating motor performance in brain injury, and that elevated levels of ET and glutamate correlate with the degree of ischemic brain injury, and are positively associated with the infarct volume.

For evidences obtained from animal model trials (56), the inhibition of the release of pro-inflammatory factor TNF-α was found to be effective in ameliorating neurological damage after brain injury. Hou et al. reported that hyperbaric oxygen rehabilitation in post-operative patients with brain tumors inhibited the expression of serum TNF-α, reduced cerebral arterial flow velocity, and effectively reduced the incidence of cerebral arterial spasm, thereby facilitating the patient’s clinical recovery (57). The present meta-analysis results for the included studies revealed that the level of serum TNF-α can be significantly reduced after stroke rehabilitation, but the change in its concentration was not found to be significantly correlated with functional improvement after stroke.

For peripheral blood biomarkers, it was found that the concentrations of nutritional indicators (ALB and HB) and antioxidant markers (CAT and SOD) significantly increased with the rehabilitation of stroke. For ALB and HB, Zhou et al. reported that low serum ALB levels can be used to predict the poor prognosis of patients with acute ischemic stroke, and that the decrease in serum ALB levels is negatively correlated with disability and mortality (58). In a retrospective study, it was found that low HB levels in stroke patients are associated with poor prognosis (59). Furthermore, the decrease in serum ALB levels may increase the risk of venous thromboembolism and pneumonia (60, 61), thereby affecting functional recovery. In contrast, elevated HB levels may improve the atherosclerosis, thereby promoting recovery (62). For CAT and SOD, since oxidative stress and hypoxia additively or synergistically exacerbate greater atherosclerosis, the increase in antioxidant enzyme activity may reduce free-radical-induced damage, and provide protection against neurological injury (63, 64).

These present findings suggest that eight biomarkers are associated with the significant functional improvement in stroke patients, while 101 biomarkers did not yield sufficient data for the meta-analysis. Over time, more data on biomarkers in stroke patients and the recovery of patients from different dysfunctions are needed to establish a timeline of biomarker changes and functional recovery progress, and determine the most sensitive and specific prognostic and diagnostic marker. In the future, brain injury biomarkers may also be normalized in brain-injured patients through therapies, such as adjunctive neuroprotective therapies, thereby improving neurological outcomes (65). The present study contained RCTs based on rigorous criteria, and the summarized evidence provided high quality data for this field.

The present study had some limitations. First, although 50 qualified RCTs were identified, focus was given on the quantitative summary of 37 articles in selecting biomarkers with datasets of ≥3. The in-depth analysis of studies with marker datasets of <3 would unlikely change the main conclusions. Furthermore, it is possible that valid biomarkers were included in the studies (Supplementary Table S3), which are not presently available from the population. Therefore, future RCTs are needed, in order to provide stronger evidence for these biomarkers. Second, the heterogeneity of the study design (e.g., type of stroke dysfunction, demographic characteristics, rehabilitation treatment approach, and biomarker measures) led to the high heterogeneity of the quantitative results. Due to the small number of biomarker studies included in the present meta-analysis, it was impossible to determine the best diagnostic marker for the functional recovery of different functional disorders, different stroke periods, and different treatment modalities. Finally, some samples of important stroke markers were identified. Therefore, future RCTs are needed to verify the robustness of the present results. Furthermore, in order to improve the replicability of the evidence, studies with a larger sample size are needed in the future. Moreover, researchers should consider conducting multi-center studies with a large sample size in the same region, in order to record the biomarker results of patients in different periods after onset and recovery from different functional impairments. In addition, in future studies, researchers should note that due to the ceiling effect and floor effect of the scale, changes in biomarkers can be used to predict whether a patient’s function tends to improve or deteriorate. This would help broaden the treatment thinking of patients with chronic stroke or severe disease.

## Conclusion

The present study was the first to conduct a meta-analysis of the influence of rehabilitation on biomarkers in stroke patients, which is correlated to functional improvement after stroke. The present results revealed that stroke rehabilitation can significantly increase the concentrations of serum BDNF, serum NE, peripheral blood SOD, peripheral blood ALB, peripheral blood HB, and peripheral blood CAT, and significantly decrease the concentrations of serum ET, serum glutamate, and serum TNF-α. In addition to serum TNF-α, the changes in other biomarkers were also associated with the significant improvement in post-stroke function. It was also revealed that serum BNDF can be used as a biomarker for NIBS treatment, and in predicting the improvement in stroke function.

## Data availability statement

The original contributions presented in the study are included in the article/Supplementary material, further inquiries can be directed to the corresponding authors.

## Author contributions

GC and MW: conceived the review and wrote the manuscript. GC, MW, JC, CZ, and QL: researched the literature. YL revised the manuscript for intellectual content. All authors contributed to the article and approved the submitted version.

## Funding

This work was supported by grant 2022YFC2009700 from Natural Key Research and Development Program of China (to YL), grant 81974357 from National Science Foundation of China (to YL), grant 202206010197 from Guangzhou Municipal Science and Technology Program (to YL), and grant 82072548 from National Science Foundation of China (to GX).

## Conflict of interest

The authors declare that the research was conducted in the absence of any commercial or financial relationships that could be construed as a potential conflict of interest.

## Publisher’s note

All claims expressed in this article are solely those of the authors and do not necessarily represent those of their affiliated organizations, or those of the publisher, the editors and the reviewers. Any product that may be evaluated in this article, or claim that may be made by its manufacturer, is not guaranteed or endorsed by the publisher.
