# Supplementary material for: Biomarkers associated with functional improvement after stroke rehabilitation: a systematic review and meta-analysis of randomized controlled trials
Source: Front Neurol. 2023 Sep 5;14:1241521. doi: 10.3389/fneur.2023.1241521 (PMC10508962; doi:10.3389/fneur.2023.1241521)
Supplement: Supplementary file 1 [file Data_Sheet_1.docx]

| **Supplementary Table 1** Search engine and query | | | | | | | | | | | | |
| --- | --- | --- | --- | --- | --- | --- | --- | --- | --- | --- | --- | --- |
| Search engine | Search query | | | | | | | | | | | Date of search |
| Pubmed (medline) | (((((stroke[MeSH Terms]) OR (stroke)) OR (cerebrovasular accident)) AND ((Rehabilitation[MeSH Terms]) OR (Rehabilitation))) AND ((((((((blood[MeSH Terms]) OR (blood)) OR (cerebrospinal fluid[MeSH Terms])) OR (cerebrospinal fluid)) OR (plasma[MeSH Terms])) OR (plasma)) OR (serum[MeSH Terms])) OR (serum))) AND (((randomized controlled trial[Publication Type]) OR (controlled clinical trial[Publication Type]) OR (randomized[Title/Abstract]) OR (placebo[Title/Abstract]) OR (randomly[Title/Abstract]) OR (trial[Title/Abstract]) OR (groups[Title/Abstract]))) | | | | | | | | | | | 8-Decemeber-22 |
|  |  |  |  |  |  |  |  |  |  |  |  |  |
|  |  |  |  |  |  |  |  |  |  |  |  |  |
|  |  |  |  |  |  |  |  |  |  |  |  |  |
|  |  | | | | | | | | | | |  |
| Embase | (stroke OR (cerebrovascular AND accident)) AND rehabilitation AND (blood OR (cerebrospinal AND fluid) OR plasma OR serum) AND ('randomized controlled trial':it OR 'controlled clinical trial':it OR randomized:ab,ti OR placebo:ab,ti OR randomly:ab,ti OR trial:ab,ti OR groups:ab,ti) | | | | | | | | | | | 8-Decemeber-22 |
|  |  |  |  |  |  |  |  |  |  |  |  |  |
|  |  |  |  |  |  |  |  |  |  |  |  |  |
|  |  |  |  |  |  |  |  |  |  |  |  |  |
|  |  |  |  |  |  |  |  |  |  |  |  |  |
|  |  |  |  |  |  |  |  |  |  |  |  |  |
| Web of science | ((TS=(stroke OR cerebrovascular accident)) AND TS=(Rehabilitation) AND TS=(blood OR cerebrospinal fluid OR plasma OR serum) AND TS=(randomized controlled trial OR controlled clinical trial OR randomized OR placebo OR randomly OR trial OR groups)) | | | | | | | | | | | 8-Decemeber-22 |
|  |  |  |  |  |  |  |  |  |  |  |  |  |
|  |  |  |  |  |  |  |  |  |  |  |  |  |

| **Supplementary Table 2** Characteristics of included studies | | | | | | | | | | | |  |
| --- | --- | --- | --- | --- | --- | --- | --- | --- | --- | --- | --- | --- |
| **References** | **Country/**  **District** | **No. of participants (Exp/Ctr)** | **Mean age (years) (Exp/Ctr)** | **Sex**  **(M/F)** | **Type of Stroke**  **(H/I)** | **Months since**  **Onset**  **(Exp/Ctr)** | **Rehabilitation prescription** | **Combined training /**  **practice** | **Purpose of rehabilitation** | **Biomarker measure (Biofluid, Quantification method)** | **Functional measurement** | |
| Bai et al (2022)(1) | China | 30/30 | 63.47/59.91 | 31/29 | H:29,  I:28,  Mix:3 | 3.27/3.75 | rTMS,80%MT,1hz,1000pulses,5d/w,4w, rIFG | Traditional speech training for 30min | Improve speech function | BDNF (serum, ELISA) | Aphasia quotient | |
| Bai et al (2021)(2) | China | 10/10/10 | 45.3 | 13/17 | H:11,  I:19 | 3 | Exp1: rTMS, 80%MT, 1hz, 1000pulses, 5d/w, 4w, rIFG  Exp2: rTMS, 80%MT, 1hz, 1000pulses, bid, 5d/w, 4w, rIFG | Traditional speech training for 20min | Improve speech function | BDNF (serum, ELISA) | Aphasia quotient | |
| Bintang et al (2020)(3) | Indonesia | 14/13 | 54.50/62.15 | 14/13 | I:27 | 40.43d/45.62d | rTMS, Bilateral M1, 1200pulses, 5d/w, 2w | standard  ischemic stroke therapy | Improve motor function | BDNF (serum, ELISA) | Stroke Rehabilitation Assessment of Movement (STREAM) | |
| Carr et al (2003)(4) | America | 20/20 | 30-82 | 22/18 | NA | ＜6 | strength training, 3d/w, 16w | Aerobic exercise for 20 minutes | NA | Glu (serum, NA);  TC (serum, NA);  HDL (serum, NA) |  | |
| Cichon et al (2018)(5) | Poland | 25/23 | 48.0/44.8 | 26/22 | NA | 3.2w/3.9w | ELF-EMF, 5d/w, 2w | Routine training 90min, Psychotherapy 15min | Improve function | BDNF (plasma, ELISA);  VEGF (plasma, ELISA) |  | |
| Cichon et al (2017)(6) | Poland | 23/34 | 68.0/70.9 | 37/20 | NA | 1.8w/2.1w | ELF-EMF,10d | Routine training 90min, Psychotherapy 15min | Improve function | CAT (blood, ELISA)  SOD (blood, ELISA) | Activities of Daily Living | |
| Faulkner et al (2013)(7) | New Zealand | 30/30 | 68/69 | 31/29 | TIA | ＜2w | Exercise training 90 min, 2d/w, 8w | None | Improve function | Glu (blood, NA);  TC (blood, NA);  HDL (blood, NA) |  | |
| Gambassi et al (2019)(8) | Brazil | 11/11 | 66.4/60.5 | 9/13 | NA | 6.6y/4.9y | strength training, 2d/w, 8w | Routine training | Improve function | CAT (plasma, NA)  SOD (plasma, NA) | Time up and go test | |
| Gjellesvik et al (2020)(9) | Norway | 36/34 | 57.6/58.7 | 41/29 | H:13，I:57 | 254.4/27.4 | HIIT, 3d/w, 8w | None | Increase or maintain peak oxygen uptake | TC (blood, NA);  HDL (blood, NA);  LDL (blood, NA);  TG (blood, NA);  HB (blood, NA) | Peak test  VO2 (L/min-1) | |
| He et al (2022)(10) | China | 30/30 | 46.29/45.43 | 39/21 | I:60 | 2w-6m | Hufu copper scraping, 1d/w, 8w | Routine training | Improves post-stroke shoulder-hand syndrome | CGRP (serum, ELISA) |  | |
| Hsu et al (2019)(11) | Taiwan | 15/15 | 55.7/57.8 | 25/5 | I:30 | 21/23 | Exercise training 36 min, 5d/w, 4w | Routine training | Improve motor function | HB (blood, NA) | 6-min walk test | |
| Huang et al (2022)(12) | Taiwan | 17/15 | 56.8/58.5 | 24/8 | I:32 | ＞3m | Exercise training 35 min, 36d | None | Improve motor function | BDNF (plasma, ELISA) |  | |
| Krawcyk et al (2019)(13) | Denmark | 31/32 | 63.7/63.7 | 49/14 | LS:63 | ≤21d | HIIT, 5d/w, 12w | None | NA | TC (blood, NA);  HDL (blood, NA);  LDL (blood, NA);  TG (blood, NA);  VEGF (plasma, ELISA) |  | |
| Lee et al (2015)(14) | Korea | 20 | 56.3 | 15/5 | I:20 | ＞1m | EA, 1d | None | Improve endothelial function | VEGF (plasma, ELISA) |  | |
| Lennon et al (2008)(15) | Ireland | 24/24 | 59.0/60.5 | 28/20 | I:48 | 237.3w/245.3w | Exercise training, 2d/w, 10w | Routine training | Improve function | TC (serum, NA); |  | |
| Liang et al (2021)(16) | China | 36/36 | 62.74/63.14 | 42/30 | H:28, I:44 | 31.27d/31.85d | VitalStim electrical stimulation, 5d/w, 4w | Swallowing function training | Improve swallowing function | SOD (serum, Xanthine oxidase method);  5-HT (serum, ELISA);  NE (serum, ELISA) | Kubota drinking water test | |
| Liu et al (2020)(17) | China | 37/37 | 56.20/55.20 | 39/35 | NA | 3.34/3.56 | rTMS, 10hz, 80%MT, 5d/w, 2m, left PFC | None | Improve post-stroke depression | BDNF (serum, ELISA);  5-HT (serum, ELISA);  NE (serum, ELISA) | Hamilton Rating Scale for Depression | |
| Lu et al (2015)(18) | China | 19/21 | 42.5/47.3 | 25/15 | H:22, I:18 | ＞1m | rTMS, 1hz, 100%MT, DLPFC, 5d/w, 4w | 30 minutes of computer-assisted cognitive training per day | Improve memory function | BDNF (plasma, ELISA) |  | |
| MacKay-Lyons et al (2022)(19) | Canada | 94/90 | 65.9/64.3 | 121/63 | NA | 2.3/2.4 | Exercise training 60min, 2d/w, 12w | None | Reducing vascular risk factors in people after stroke | Glu (blood, NA);  HDL (blood, NA);  LDL (blood, NA);  TG (blood, NA); |  | |
| Mao et al (2022)(20) | China | 20/20 | 59.80/61.25 | 19/21 | H:2,  I:38 | 3.25/3.6 | tDCS, 6d/w, 8w | Swallowing function training | Improve swallowing function | HB (blood, NA);  ALB (blood, NA) | Functional Dysphagia Scale | |
| Qin et al (2022)(21) | China | 61/61 | 61.93/63.2 | 78/44 | H:48, I:74 | ＜3d | Acupuncture, 7d | None | Improve gastrointestinal dynamics | HB (blood, NA);  ALB (blood, NA) | motility index of gastric antrum | |
| Tang et al (2021)(22) | China | 36/36 | 69.3/68.9 | 41/31 | H:23, I:49 | 33.08d/32.64d | meridians warm needling method, 7d/w, 4w | Routine training | Improves post-stroke shoulder-hand syndrome | CGRP (serum, NA) |  | |
| Utomo et al (2020)(23) | Indonesia | 11/11 | 55.00/54.73 | 14/8 | I:22 | Subacute Phase | tDCS, 5d | Occupational Therapy | To investigate the effect of tDCS on BDNF serum in stroke patients | BDNF (serum, ELISA) | None | |
| Vahlberg et al (2021)(24) | Sweden | 40/39 | 63.9/63.9 | 50/29 | H:9, I:57, TIA:13 | NA | Regular outdoor walking and functional leg exercises, 3m | None | Improve function | TC (blood, NA);  HDL (blood, NA);  LDL (blood, NA) |  | |
| Wang et al (2021)(25) | China | 50/50 | 65.3/66.5 | 62/38 | H:44, I:56 | ＜7d | Cognitive function training 30min, 7d/w, 8w | Routine training | Improve cognitive function | BDNF (serum, ELISA);  5-HT (serum, ELISA);  NE (serum, ELISA) ;  Glutamate (serum, ELISA) | Mini-mental State Examination | |
| Wang et al (2022)(26) | China | 60/60 | NA | 59/61 | H:41, I:79 | ＜3m | Vitamin C stimulation | Swallowing function training | Improve swallowing function | HB (serum, NA);  ALB (serum, NA) | video fluoroscopic swallowing study | |
| Wang et al (2022)(27) | China | 38/39 | 67.24/68.13 | 37/40 | I:77 | ＜1w | EA, 7d/w, 2w | None | Improve function | TNF-α（serum, ELISA） | National Institute of Health stroke scale | |
| Wang et al (2022)(28) | China | 60/60 | 64.41/64.43 | 65/55 | I:120 | Acute Phase | HOT, 7d/w, 2w | None | Improve function | TNF-α (serum, ELISA);  ET (serum, ELISA) | National Institute of Health stroke scale | |
| Wang et al (2022)(29) | China | 20/20 | 61.70/60.95 | 19/21 | I:40 | 2-3m | enriched rehabilitation, 6d/w, 8w | None | Improve cognitive function | Glutamate (serum, ELISA) | Symbol Digit Modalities Test | |
| Wang et al (2022)(30) | China | 30/30 | 57.73/54.57 | 48/12 | H:24, I:36 | ＞1m | TUS, 5d/w,＞6w | Cognitive function training | Improve cognitive function | BDNF (serum, ELISA) | Mini-mental State Examination | |
| Wang et al (2021)(31) | China | 60/60 | 49y-72y | 65/55 | I:120 | ＜6m | HOT, 14d | Routine training | To investigate the effect of HOT on neurotrophic factor secretion and oxidative stress in stroke patients | CAT (blood, ELISA)  SOD (blood, ELISA)  Glutamate (serum, RIA) | None | |
| Xiong et al (2020)(32) | China | 35/35 | 63.0/65.3 | 37/33 | NA | 2.13/2.51 | scalp acupuncture, 6d/w, 8w | Routine training | Improve cognitive and motor function | BDNF (plasma, ELISA) |  | |
| Zhang et al (2013)(33) | China | 49/49 | 62.8/63.3 | 66/32 | I:98 | 1-2d | EA, 7d/w, 3w | Routine training | Improve function | ET (serum, RIA) | Barthel index | |
| Zhang et al (2021)(34) | China | 108/106 | 69.88/70.25 | 126/88 | NA | 8.39/8.53 | Mendelsohn maneuver and swallowing training,1m | Routine training | Improve swallowing function | TNF-α (serum, ELISA) | video fluoroscopic  swallowing exam | |
| Zhang et al (2022)(35) | China | 55/55 | 64.65/62.29 | 57/53 | I:110 | 2.18/2.10 | Acupuncture,14d | None | Improve function | CGRP (serum, ELISA);  ET (serum, ELISA) | National Institute of Health stroke scale | |
| Zhao et al (2021)(36) | China | 19/19 | 50.16/48.95 | 21/17 | H:21, I:17 | 2.85m | rTMS, 10hz, M1, 80%RMT, 1500pulses, 6d/w, 3w | Routine training | Improve acute neuropathic pain after stroke | BDNF (serum, ELISA) | Numeric Rating Scale | |
| Zhao et al (2022)(37) | China | 14/14 | 50.1/56.1 | 25/3 | H:14, I:14 | 47.1d/27.5d | BCI, 6d/w, 4w | Routine training | Improve function | BDNF (serum, ELISA) | Modified Barthel Index | |
| Exp, experimental group; Ctr, control group; M, male; F, female; H, Hemorrage; I, Ischemia; TIA, Transient ischemic attack; LS, lacunar stroke; RMT, resting motor threshold; MT, motor threshold; Hz, hertz; d, days; w, weeks; m, months; y, years; AH, Affected hemisphere; UH, Unaffected hemisphere; M1, primary motor cortex; rIFG, right inferior frontal gyrus; DLPFC, dorsolateral prefrontal cortex; PFC, prefrontal cortex; rTMS, repetitive transcranial magnetic stimulation; tDCS, transcranial direct current stimulation; ELF-EMF, Extremely low frequency electromagnetic field; HIIT, high-intensity interval training; EA, electroacupuncture; HOT, hyperbaric oxygen treatment; TUS, transcranial ultrasound stimulation; BCI, brain-computer interface; BDNF, brain-derived neurotrophic factor; NA, not available; Glu, glucose; TC, total cholesterol; HDL, high density lipoprotein; LDL, low density lipoprotein; TG, Triglyceride; VEGF, vascular endothelial growth factor; CAT, catalase; SOD, Superoxide Dismutase; HB, Hemoglobin; ALB, albumin ; CGRP, calcitonin-gene-related peptide; 5-HT, 5-hydroxytryptamine; NE, Noradrenaline; TNF-α, tumor necrosis factor-α; ET, Endothelin; ELISA, enzyme linked immunosorbent assay; RIA, Radio immunoassay. | | | | | | | | | | | |  |

| **Supplementary Table 3** Qualitative review table | | | | |
| --- | --- | --- | --- | --- |
| **Biomarker** | **Biofluid** | **References** | **Method** | **Rehabilitation vs Control** |
| Erythrocyte | blood | Hsu et al (2019)(11) | NA | Equivocal |
| Non-HDL-cholesterol | blood | Vahlberg et al (2021)(24) | NA | decreased ab |
| HbA1c | blood | Gjellesvik et al (2020)(9) | NA | Equivocal |
|  | blood | Vahlberg et al (2021)(24) | NA | Increased |
| C-peptide | blood | Gjellesvik et al (2020)(9) | NA | decreased |
| Lactate | blood | Gjellesvik et al (2020)(9) | NA | Increased c |
| HGF | plasma | Cichon et al (2018)(5) | Bio-Plex200 System | Increased a |
| SCF | plasma | Cichon et al (2018)(5) | Bio-Plex200 System | Increased a |
| SDF-1α | plasma | Cichon et al (2018)(5) | Bio-Plex200 System | Equivocal |
| β-NGF | plasma | Cichon et al (2018)(5) | Bio-Plex200 System | NA |
| NGF | serum | Wang et al (2021)(31) | ELISA | Increased abc |
|  | plasma | Xiong et al (2020)(32) | ELISA | Increased abc |
| LIF | plasma | Cichon et al (2018)(5) | Bio-Plex200 System | NA |
| TAS | plasma | Cichon et al (2017)(6) | ELISA | Equivocal |
| SOD1 mRNA | blood | Cichon et al (2018)(38) | Real-time  polymerase chain reaction | Increased a |
| SOD2 mRNA | blood | Cichon et al (2018)(38) | Real-time  polymerase chain reaction | Increased a |
| GPX1mRNA | blood | Cichon et al (2018)(38) | Real-time  polymerase chain reaction | Increased a |
| GPX4mRNA | blood | Cichon et al (2018)(38) | Real-time  polymerase chain reaction | Increased a |
| NOS2mRNA | blood | Cichon et al (2017)(39) | Real-time  polymerase chain reaction | decreased |
| MPO | plasma | Hsu et al (2019)(11) | ELISA | Decreased a |
|  | plasma | Huang et al (2022)(12) | ELISA | Increased |
| MMP-9 | serum | Zhao et al (2008)(40) | ELISA | Decreased abc |
| NADPH oxidase | plasma | Gambassi et al (2019)(8) | NA | Decreased |
| 3-NT | plasma | Cichon et al (2017)(39) | c-ELISA | Increased a |
| Nitrite | plasma | Gambassi et al (2019)(8) | NA | Decreased |
| H2O2 | plasma | Gambassi et al (2019)(8) | NA | Decreased |
| nitric oxide | serum | Zhang et al (2022)(35) | ELISA | Increased c |
| TNF | plasma | Krawcyk et al (2019)(13) | ELISA | Equivocal |
|  | serum | Wang et al (2022)(29) | ELISA | decreased abc |
| carbonyl | plasma | Cichon et al (2018)(41) | ELISA | Decreased ac |
|  | plasma | Gambassi et al (2019)(8) | NA | Decreased ac |
| thiol | plasma | Cichon et al (2018)(41) | ELISA | Increased ac |
| TBARS | plasma | Cichon et al (2018)(41) | ELISA | Decreased ac |
|  | plasma | Gambassi et al (2019)(8) | NA | Decreased ac |
| IL-1 | serum | Zhang et al (2021)(34) | ELISA | Decreased abc |
| IL-1β | plasma | Cichon et al (2019)(42) | ELISA | Increased ac |
| IL-1β mRNA | plasma | Cichon et al (2019)(42) | NA | Increased ac |
| IL-2 | plasma | Cichon et al (2019)(42) | ELISA | Increased ac |
| IL-4 | serum | Li et al (2021)(43) | Flow CytoMetry | Increased abc |
| IL-6 | plasma | Hsu et al (2019)(11) | ELISA | Decreased a |
|  | plasma | Krawcyk et al (2019)(13) | ELISA | Decreased a |
|  | serum | Zhang et al (2021)(34) | ELISA | Decreased abc |
| IL-10 | plasma | Lee et al (2015)(14) | ELISA | Equivocal |
| IFN-γ | plasma | Cichon et al (2019)(42) | ELISA | Increased |
| TGF-β | plasma | Cichon et al (2019)(42) | ELISA | Equivocal |
| ICAM-1 | plasma | Krawcyk et al (2019)(13) | ELISA | Decreased abc |
|  | serum | Feng et al (2019)(44) | ELISA | Decreased c |
| sICAM | serum | Zhao et al (2008)(40) | ELISA | Decreased abc |
| VCAM-1 | plasma | Krawcyk et al (2019)(13) | ELISA | Increased ac |
| sVCAM | serum | Zhao et al (2008)(40) | ELISA | Decreased abc |
| ET-1 | serum | Feng et al (2019)(44) | ELISA | Decreased c |
| substance P | serum | He et al (2022)(10) | ELISA | Decreased abc |
|  | serum | Tang et al (2021)(22) | NA | Decreased abc |
| P-selectin | plasma | Huang et al (2022)(12) | ELISA | Decreased |
| soluble E-selectin | serum | Zhao et al (2008)(40) | ELISA | Decreased abc |
| soluble protein-100B | serum | Zhang et al (2013)(33) | automated immunoluminometric  assay | Decreased abc |
| s-100 | serum | Zhang et al (2022)(35) | fluorescence immunoassay | Decreased c |
| E-selectin | plasma | Krawcyk et al (2019)(13) | ELISA | Decreased |
| Hematocrit | blood | Tang et al (2021)(22) | NA | Decreased abc |
|  | blood | Hsu et al (2019)(11) | NA | Equivocal |
| Leukocyte | blood | Hsu et al (2019)(11) | NA | Equivocal |
| Platelet | blood | Hsu et al (2019)(11) | NA | Equivocal |
| MIP-1β | plasma | Huang et al (2022)(12) | ELISA | Decreased a |
| tPA | plasma | Huang et al (2022)(12) | ELISA | Increased |
| PAI-1 | plasma | Huang et al (2022)(12) | ELISA | Decreased |
| vWF | plasma | Huang et al (2022)(12) | ELISA | Increased |
| Lp-PLA2 | blood | Huang et al (2022)(12) | NA | Decreased |
| ADMA | blood | Huang et al (2022)(12) | NA | Equivocal |
| Mono1 | blood | Huang et al (2022)(12) | Flow CytoMetry | Equivocal |
| Mono2 | blood | Huang et al (2022)(12) | Flow CytoMetry | Equivocal |
| Mono3 | blood | Huang et al (2022)(12) | Flow CytoMetry | Equivocal |
| MPA | blood | Huang et al (2022)(12) | Flow CytoMetry | Decreased ac |
| MPA1 | blood | Huang et al (2022)(12) | Flow CytoMetry | Decreased a |
| MPA2 | blood | Huang et al (2022)(12) | Flow CytoMetry | Decreased a |
| MPA3 | blood | Huang et al (2022)(12) | Flow CytoMetry | Decreased |
| thrombin generation | blood | Huang et al (2022)(12) | NA | Equivocal |
| TLR4 | serum | Ji et al (2020)(45) | ELISA | Decreased c |
| NF-κB | serum | Ji et al (2020)(45) | ELISA | Decreased c |
| Pro-ADM | plasma | Krawcyk et al (2019)(13) | ELISA | Increased a |
| Pro-ANP | plasma | Krawcyk et al (2019)(13) | ELISA | Increased a |
| Copeptin | plasma | Krawcyk et al (2019)(13) | ELISA | Equivocal |
| Insulin | plasma | Krawcyk et al (2019)(13) | ELISA | Decreased bc |
| Insulin- like growth factor-1 | serum | Vahlberg et al (2021)(24) | NA | Decreased |
| EPCs | blood | Lee et al (2015)(14) | Flow CytoMetry | Increased ac |
|  | plasma | Zhen et al (2016)(46) | ELISA | Increased abc |
| T3 | serum | Li et al (2021)(47) | ECLI | Increased abc |
| T4 | serum | Li et al (2021)(47) | ECLI | Decreased |
| FT3 | serum | Li et al (2021)(47) | ECLI | Increased abc |
| FT4 | serum | Li et al (2021)(47) | ECLI | Decreased |
| TSH | serum | Li et al (2021)(47) | ECLI | Increased ac |
| MDA | serum | Liang et al (2021)(16) | ELISA | Decreased abc |
|  | serum | Wang et al (2022)(29) | thiobarbituric acid  reagent | Decreased abc |
| NPY | serum | Wang et al (2022)(28) | ELISA | Decreased abc |
|  | serum | Liu et al (2020)(17) | ELISA | Increased abc |
| NT-proBNP | serum | Zhang et al (2022)(35) | immunoradiometric assay | Decreased c |
| CRF | serum | Liu et al (2020)(17) | ELISA | Decreased abc |
| DA | serum | Liu et al (2020)(17) | ELISA | Decreased ab |
| epinephrine | serum | Liu et al (2020)(17) | ELISA | Decreased ab |
| white blood cell | blood | Mao et al (2022)(20) | NA | Decreased c |
| Prealbumin (PAB) | blood | Mao et al (2022)(20) | NA | Increased |
|  | blood | Qin et al (2022)(21) | NA | Increased c |
| c-reactive protein | blood | Mao et al (2022)(20) | NA | Decreased c |
| total protein | serum | Wang et al (2022)(26) | NA | Increased abc |
| MBP | serum | Wang et al (2021)(31) | ELISA | Decreased abc |
| NSE | serum | Wang et al (2021)(31) | ELISA | Decreased abc |
|  | serum | Zhang et al (2013)(33) | immunofluorometric assay | Decreased abc |
| GABA | serum | Wang et al (2021)(31) | ELISA | Increased abc |
| ROS | serum | Wang et al (2021)(31) | ELISA | Decreased abc |
| LHP | serum | Wang et al (2021)(31) | ELISA | Decreased abc |
| lgA | blood | Wang et al (2022)(26) | immunoscattering turbidimetric method | Increased abc |
| lgM | blood | Wang et al (2022)(26) | immunoscattering turbidimetric method | Increased abc |
| lgG | blood | Wang et al (2022)(26) | immunoscattering turbidimetric method | Increased abc |
| TNF-α | plasma | Cichon et al (2017)(39) | ELISA | Equivocal |
| BAP | serum | pang et al (2013)(48) | ELISA | Equivocal |
| CTx | serum | pang et al (2013)(48) | ELISA | Equivocal |
| VEGF | serum | Zhen et al (2016)(46) | ELISA | Increased ac |
|  | serum | Zheng et al (2018)(49) | ELISA | Increased ab |
| SS | CSF | Zhang et al (1999)(50) | ELISA | Increased |
| HGF, hepatocyte growth factor; SCF, stem cell factor; SDF-1α, stromal cell-derived factor 1α; β-NGF, nerve growth factor β; LIF, leukemia inhibitory factor; TAS, total antioxidant status; SOD, superoxide dismutase; GPX, glutathione peroxidase; NOS, nitric oxide synthase; TNF, tumor necrosis factor; 3-NT, 3-nitrotyrosine; TBARS, thiobarbituric acid reactive substances; IL, interleukin; INFγ, interferon γ; ICAM-1, intercellular adhesion molecule-1; ET-1, endothelin-1; H2O2, Nitrite peroxide; HbA1c, glycosylated hemoglobin; MPO, myeloperoxidase; MIP-1β, Macrophage inflammatory protein 1β; tPA, tissue plasminogen activator; PAI-1, plasminogen activator inhibitor-1; vWF, von-Willebrand factor; Lp-PLA, lipoprotein-associated phospholipase A2; ADMA, asymmetric dimethylarginine; MPA, monocyte-platelet aggregates; TLR4, Toll-like receptors 4; NF-κB, Nuclear factor kappa-B; Pro-ADM, Pro-adrenomedullin; Pro-ANP, Pro-atrial natriuretic peptide; ICAM-1, intercellular adhesion molecule-1; VCAM-1, vascular cell adhesion molecule-1; EPCs, endothelial progenitor cells; T3, triiodothyronine; T4, total serum thyroxine; FT3, free triiodothyronine; FT4, free thyroxine; TSH, thyroid stimulating hormone; MDA, malondialdehyde; NPY, nerve peptide Y; CRF, corticotropin releasing factor; DA, dopamine; BAP, bone-specific alkaline phosphatase; CTx, C-telopeptide of type I collagen cross-links; HDL, high density lipoprotein; NPY, neuropeptide Y; NGF, nerve growth factor; MBP, myelin basic protein; NSE, neuron specific enolase; ROS, reactive oxygen species; LHP, lipid hydrogen peroxide; GABA, γ-aminobutyric acid; sICAM, soluble intercellular adhesion molecule; sVCAM, soluble vascular cell adhesion molecule; MMP-9: matrix metalloproteinase; VEGF, vascular endothelial growth factor; TGF-β,transforming growth factor-β; ELISA, enzyme linked immunosorbent assay; NA, not available; ECLI, electrochemiluminescence immunoassay.  a There was a significant pre and post intervention within the rehabilitation group (p < 0.05).  b There was a significant pre and post intervention within the control group (p < 0.05).  c Significant values in the rehabilitation group versus the control group after treatment (p < 0.05) | | | | |

| **Supplementary Table 4**  Grade (summary of findings) | | | | | | |
| --- | --- | --- | --- | --- | --- | --- |
| **rehabilitation group compared to control group for stroke patients**  **Patient or population:** stroke patients **Settings:**  **Intervention:** rehabilitation group **Comparison:** control group | | | | | | |
| **Outcomes** | **Illustrative comparative risks* (95% CI)** | | **Relative effect (95% CI)** | **No of Participants (studies)** | **Quality of the evidence (GRADE)** | **Comments** |
|  | Assumed risk | Corresponding risk |  |  |  |  |
|  | **Control group** | **rehabilitation group** |  |  |  |  |
| **Serum BDNF** |  | The mean concentration in the intervention groups was **1.57 standard deviations higher** (0.70 to 2.44) |  | 431 (10 studies) | ⊕⊕⊕⊝ **Moderate**^1^ | SMD 1.57 (0.70 to 2.44) |
| **Serum ET** |  | The mean concentration in the intervention groups was **2.29 standard deviations** **lower** (4.48 to 0.10 lower) |  | 328 (3studies) | ⊕⊕⊝⊝  **Low** ^1,2^ | SMD -2.29 (-4.48 to -0.10) |
| **Serum NE** |  | The mean concentration in the intervention groups was **0.94 standard deviations higher** (0.33 to 1.54 ) |  | 246 (3 studies) | ⊕⊕⊝⊝  **Low** ^1,2^ | SMD 0.94 (0.33 to 1.54) |
| **Serum Glutamate** |  | The mean concentration in the intervention groups was **0.92 standard deviations higher** (1.34 to 0.51 lower) |  | 260 (3 studies) | ⊕⊕⊝⊝  **Low** ^1,2^ | SMD -0.92 (-1.34 to -0.51) |
| **Blood SOD** |  | The mean concentration in the intervention groups was **4.17 standard deviations higher** (1.52 to 6.82) |  | 271 (4 studies) | ⊕⊕⊝⊝  **Low** ^1,2^ | SMD 4.17 (1.52 to 6.82) |
| **Blood ALB** |  | The mean concentration in the intervention groups was **1.45 standard deviations higher** (0.31 to 2.58) |  | 282 (3 studies) | ⊕⊕⊝⊝  **Low** ^1,2^ | SMD 1.45 (0.31 to 2.58) |
| **Blood HB** |  | The mean concentration in the intervention groups was **1.62 standard deviations higher** (0.62 to 2.62) |  | 374 (5 studies) | ⊕⊕⊝⊝  **Low** ^1,2^ | SMD 1.62 (0.62 to 2.62) |
| **Blood CAT** |  | The mean concentration in the intervention groups was **11.87 standard deviations higher** (5.98 to 17.76) |  | 199 (3 studies) | ⊕⊕⊝⊝  **Low** ^1,2^ | SMD 11.87 (5.98 to 17.76) |
| *The basis for the **assumed risk** (e.g., the median control group risk across studies) is provided in footnotes. The **corresponding risk** (and its 95% confidence interval) is based on the assumed risk in the comparison group and the **relative effect** of the intervention (and its 95% CI). **CI:** Confidence interval; **BDNF,** brain-derived neurotrophic factor**;** **ET,** Endothelin**; NE,** Noradrenaline**;** **SOD,** Superoxide Dismutase**; ALB,** albumin **; HB,** Hemoglobin**; CAT,** catalase**;** | | | | | | |
| GRADE Working Group grades of evidence **High quality:** Further research is very unlikely to change our confidence in the estimate of effect.  **Moderate quality:** Further research is likely to have an important impact on our confidence in the estimate of effect and may change the estimate. **Low quality:** Further research is very likely to have an important impact on our confidence in the estimate of effect and is likely to change the estimate. **Very low quality:** We are very uncertain about the estimate. | | | | | | |
| ^1^ Serious inconsistency due to moderate-severe heterogeneity with 50% < I ^2^ < 100% and P value (chi-square test) < 0.05. ^2^ Very serious imprecision due to the small sample size (< 400 individuals) and wide confidence interval | | | | | | |


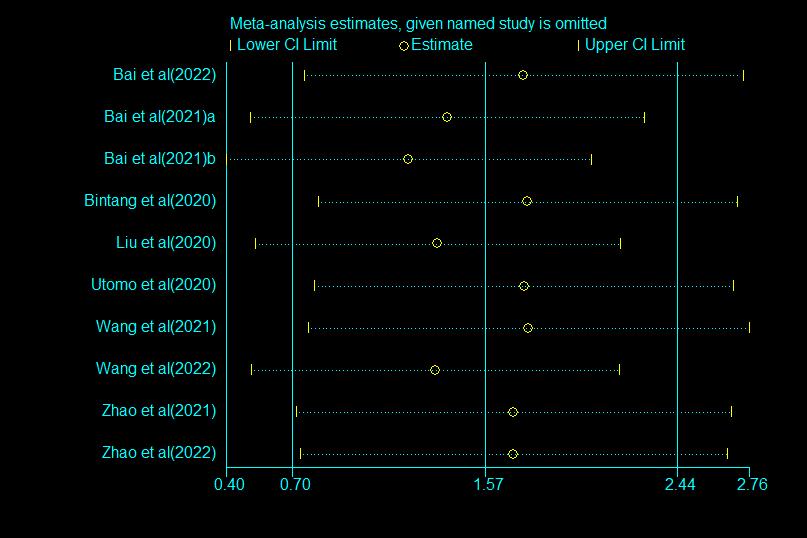


**Supplementary Figure 1** Results of sensitivity analysis showing the stability of the results in the included studies


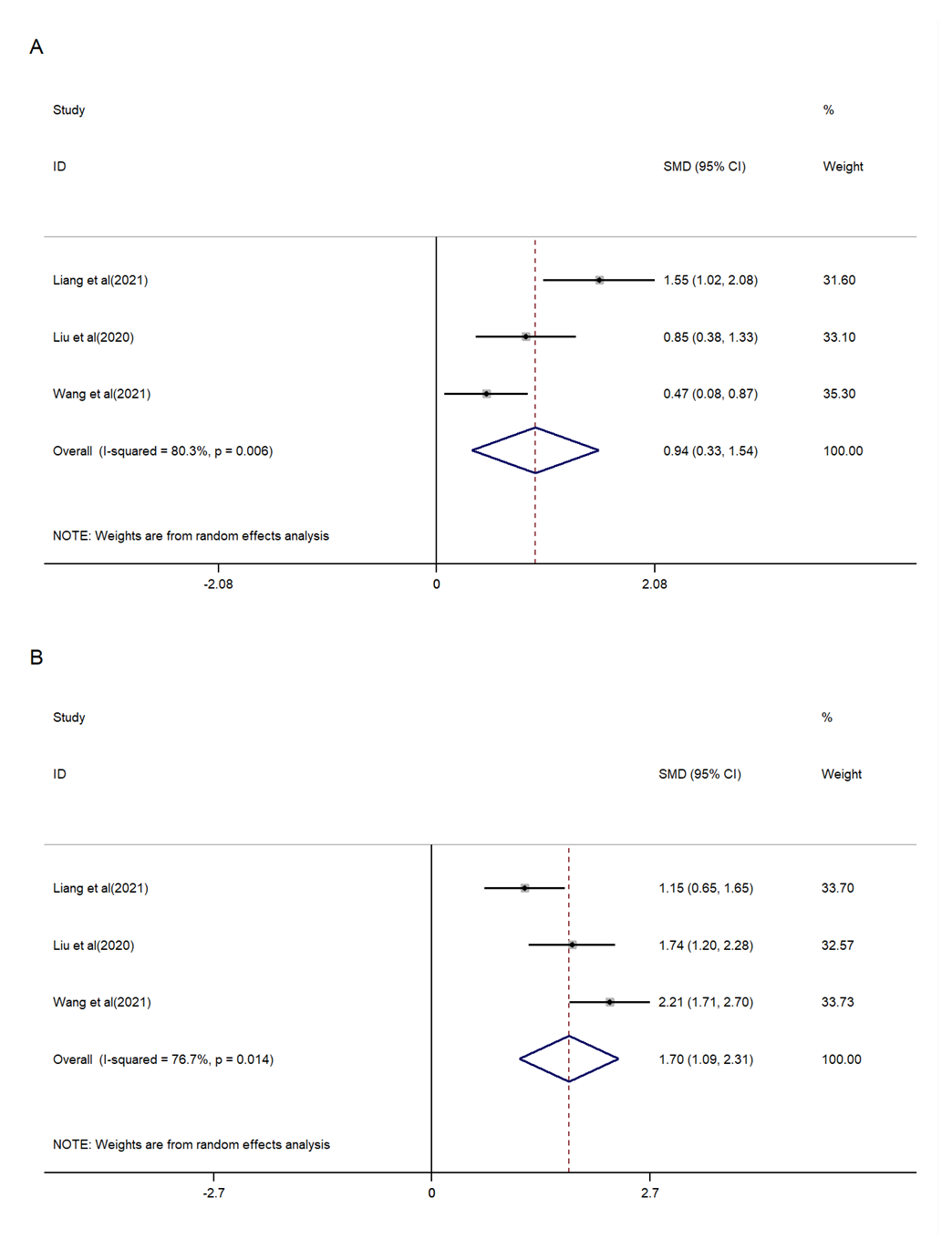


**Supplementary Figure 2** A. Forest plot of the effect of rehabilitation treatment on serum Noradrenaline (NE) in stroke patients; B. Forest plot of the effect of rehabilitation therapy on functional recovery in stroke patients in the Serum NE Study.


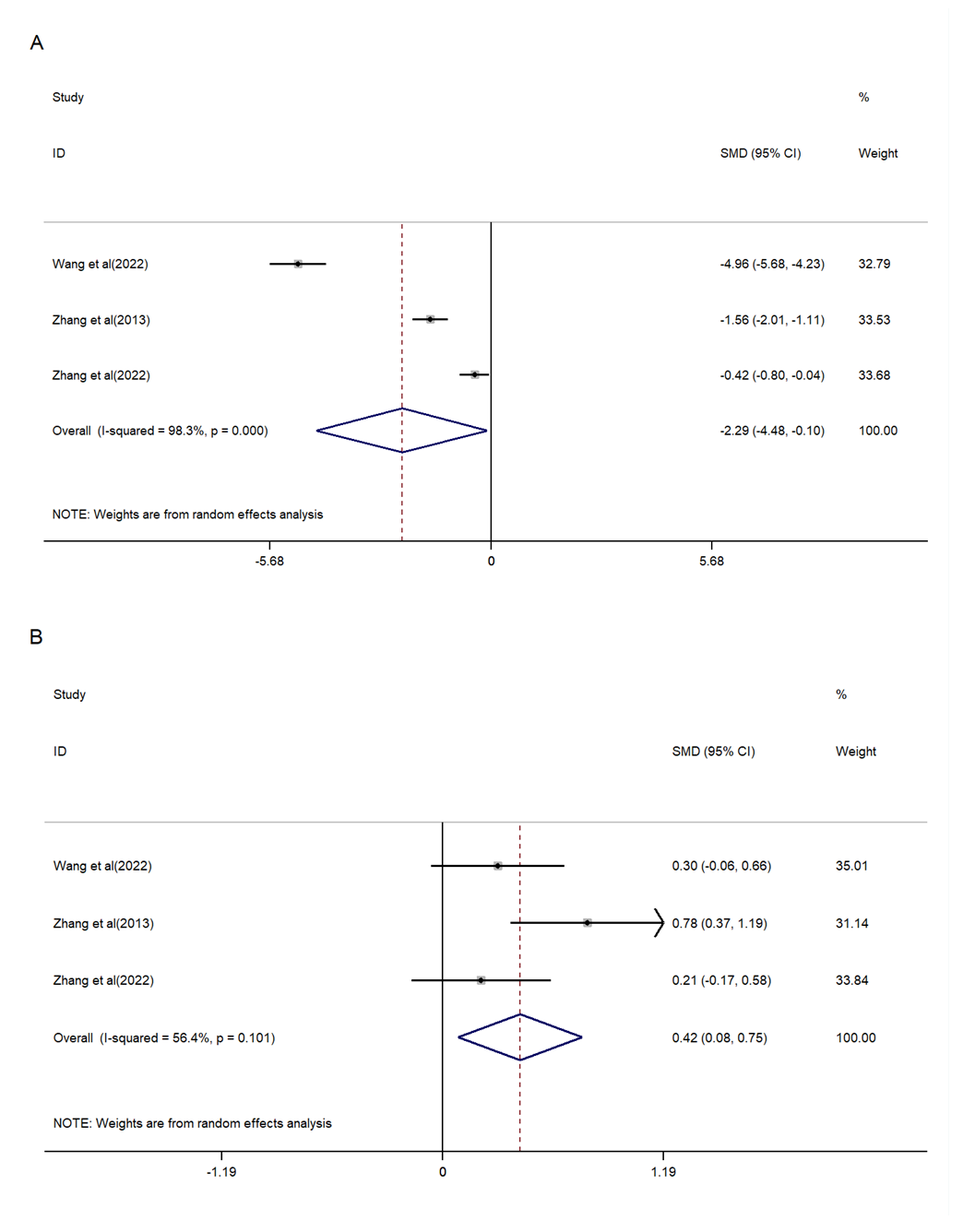


**Supplementary Figure 3** A. Forest plot of the effect of rehabilitation treatment on serum Endothelin (ET) in stroke patients; B. Forest plot of the effect of rehabilitation therapy on functional recovery in stroke patients in the Serum ET Study.


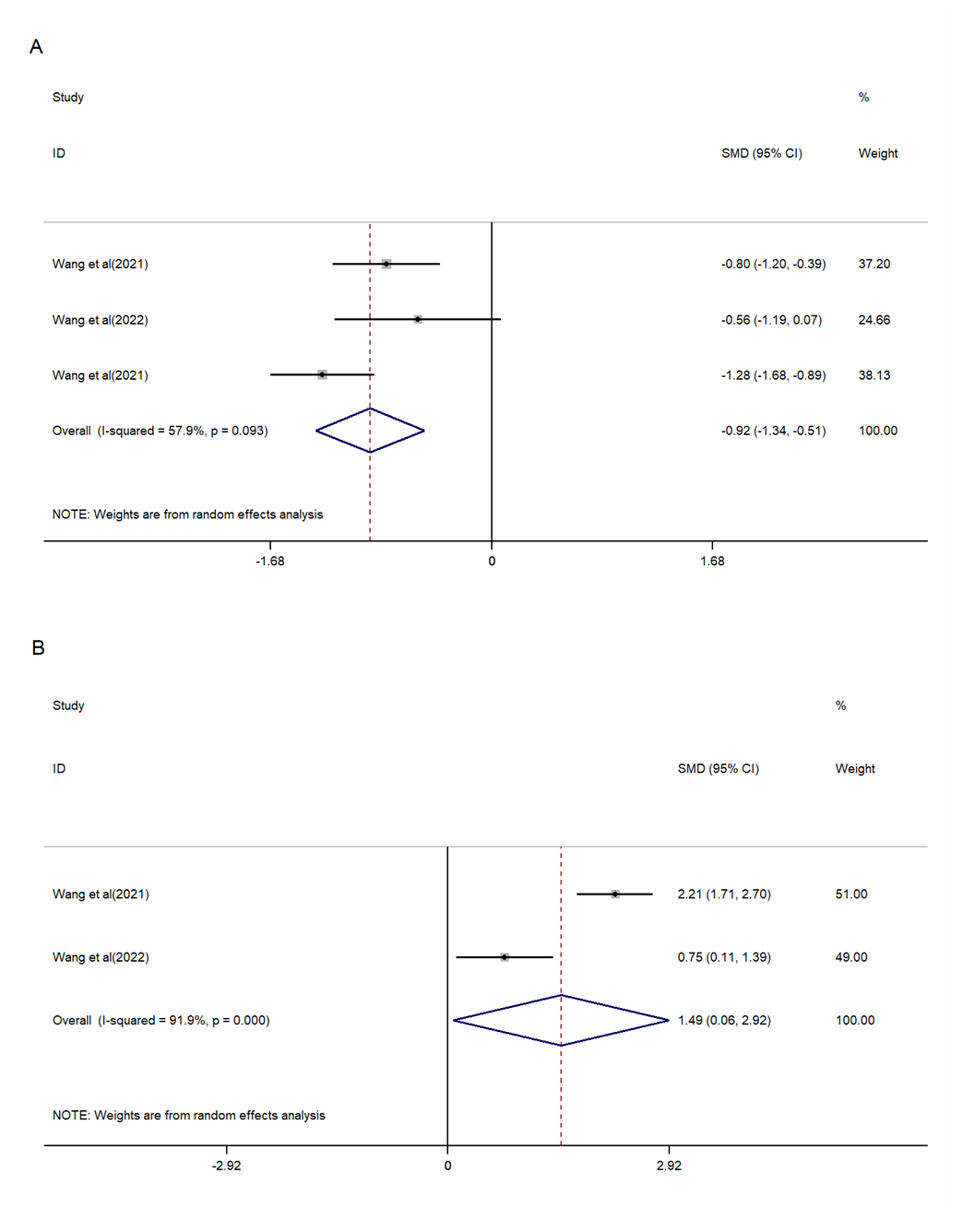


**Supplementary Figure 4** A. Forest plot of the effect of rehabilitation treatment on serum Glutamate in stroke patients; B. Forest plot of the effect of rehabilitation therapy on functional recovery in stroke patients in the Serum Glutamate Study.


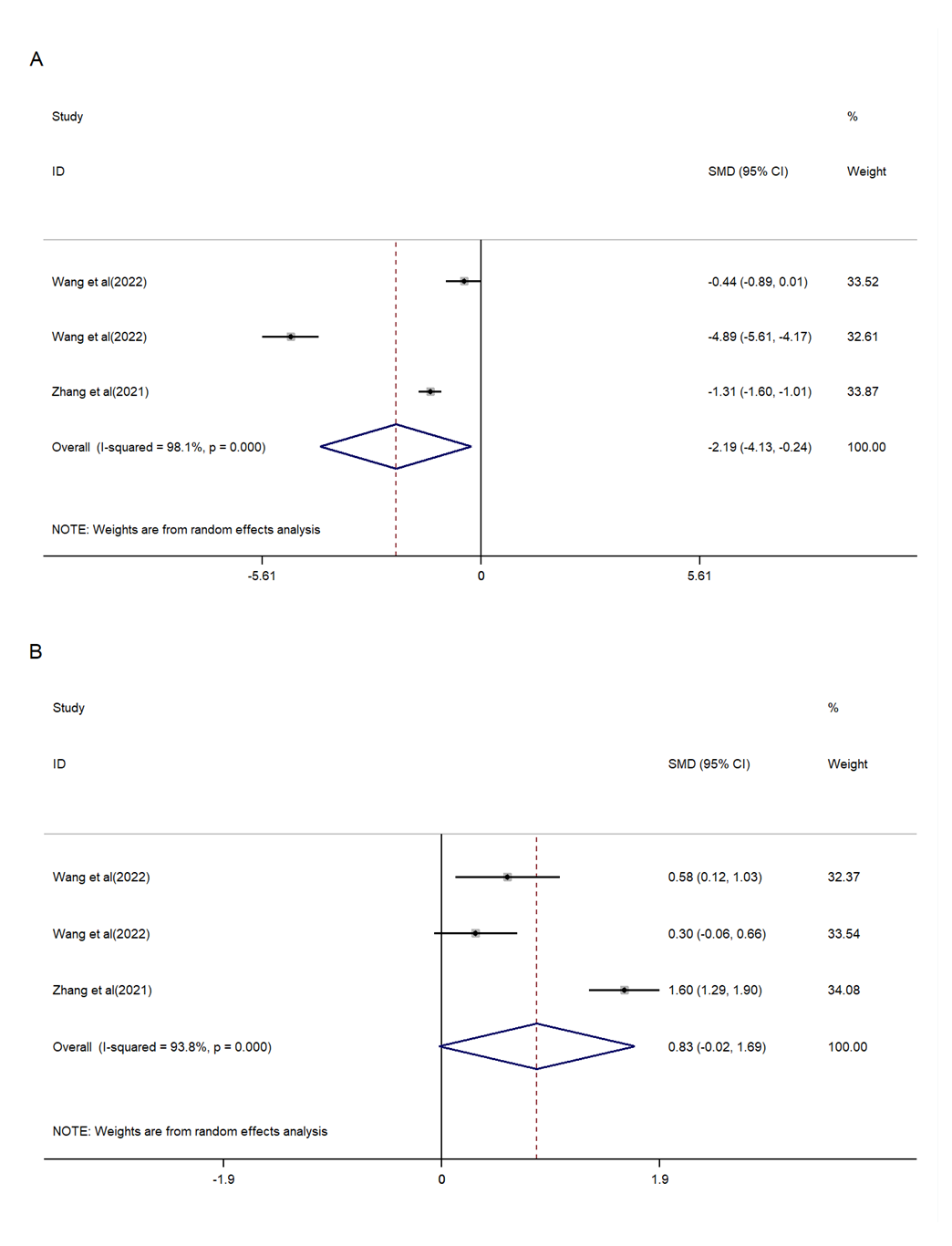


**Supplementary Figure 5** A. Forest plot of the effect of rehabilitation treatment on serum tumor necrosis factor-α (TNF-α) in stroke patients; B. Forest plot of the effect of rehabilitation therapy on functional recovery in stroke patients in the Serum TNF-α Study.


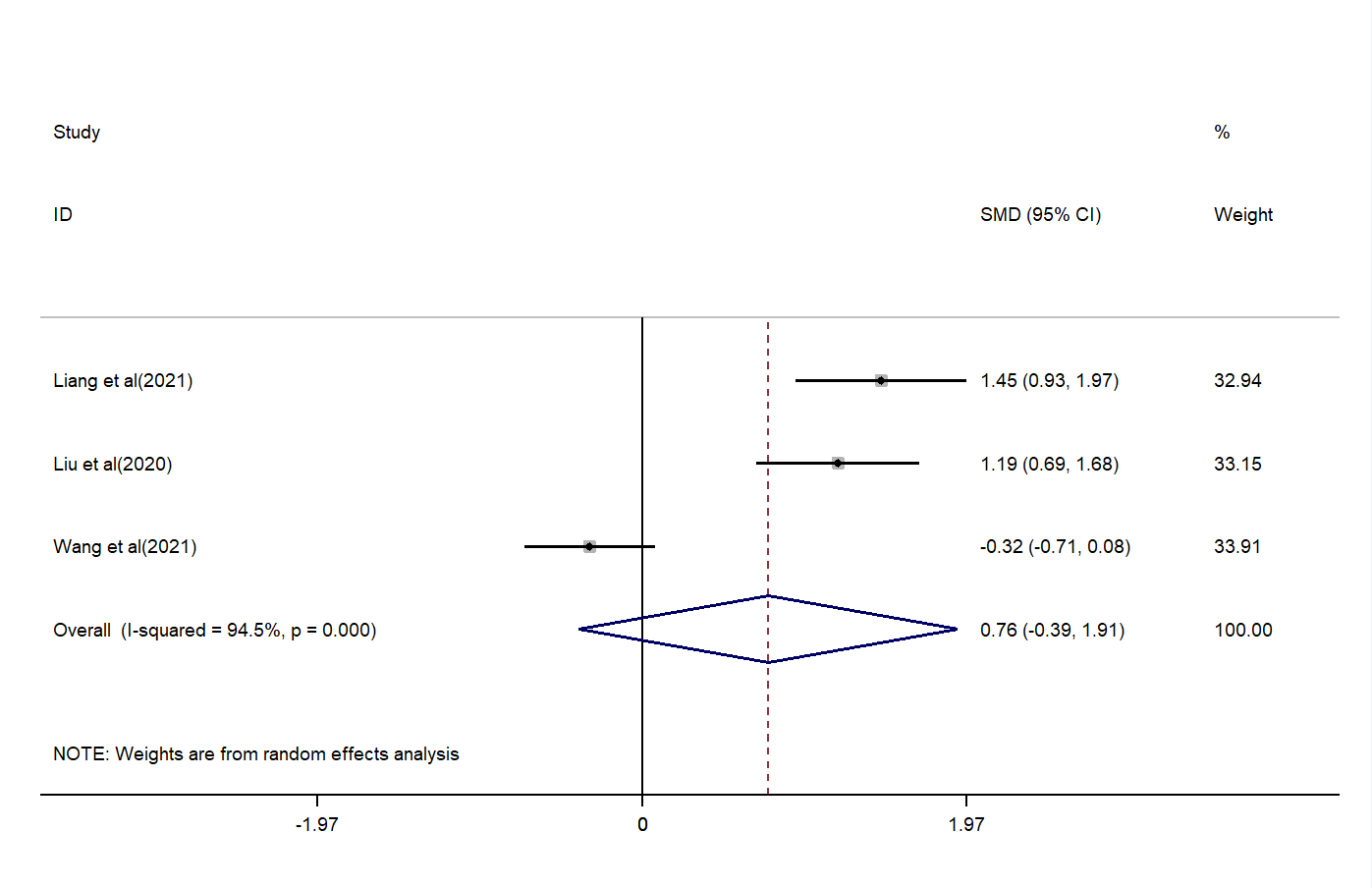


**Supplementary Figure 6** Forest plot of the effect of rehabilitation treatment on serum 5-hydroxytryptamine in stroke patients.


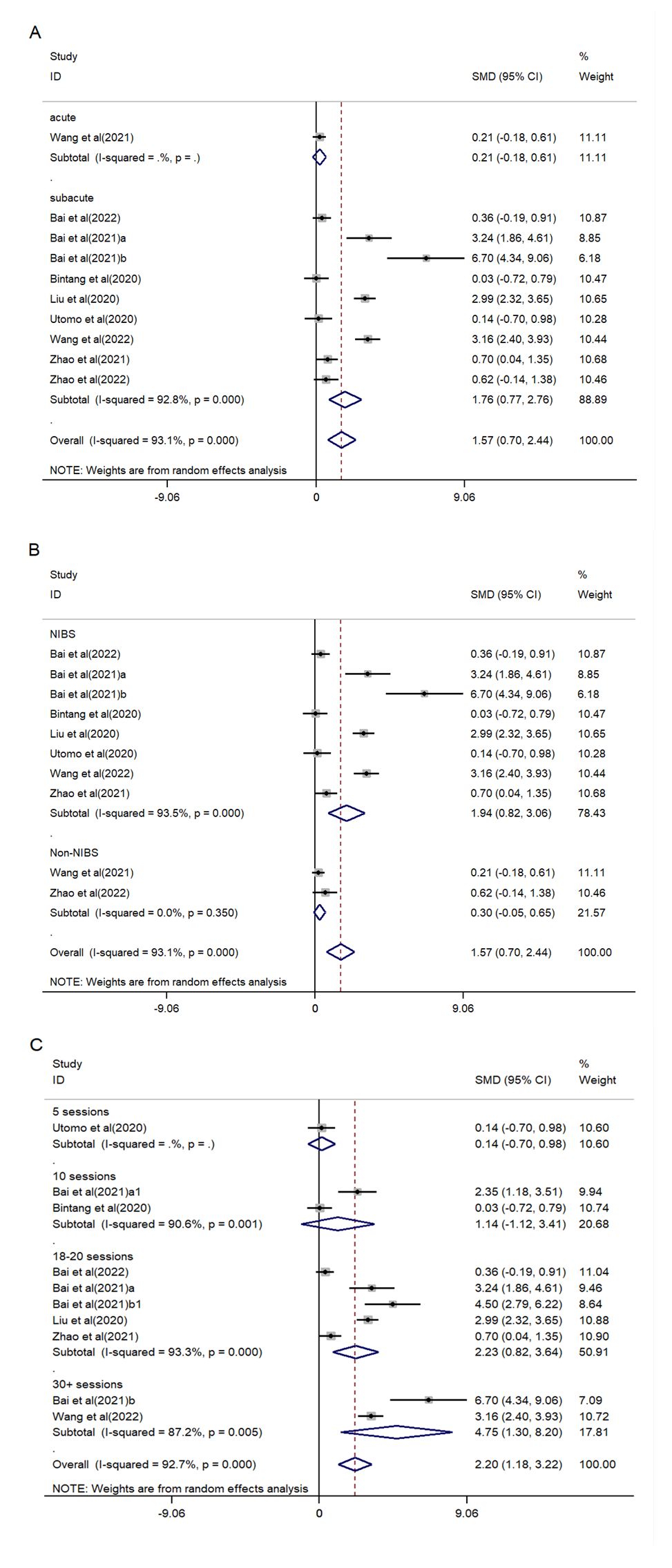


**Supplementary Figure 7** A. Forest plot of serum brain-derived neurotrophic factor (BDNF) concentration changes in stroke patients disaggregated by Stage of stroke compared with controls; B. Forest plot of serum BDNF concentration changes in stroke patients disaggregated by Rehabilitation method compared with controls; C. Forest plot of serum BDNF concentration changes in stroke patients disaggregated by Treatment sessions (non-invasive brain stimulation, NIBS) compared with controls


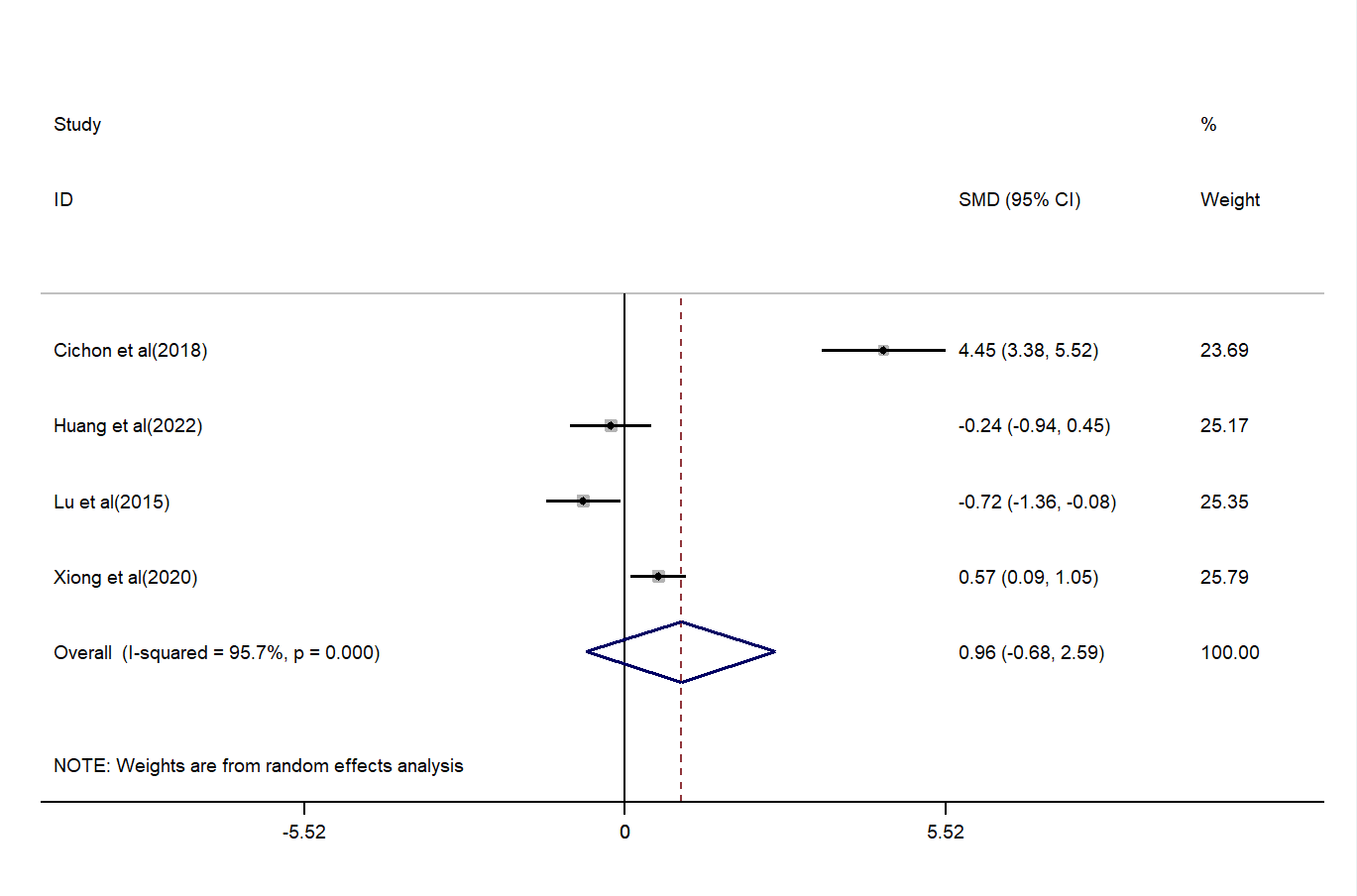


**Supplementary Figure 8** Forest plot of the effect of rehabilitation treatment on plasma brain-derived neurotrophic factor (BDNF) in stroke patients.


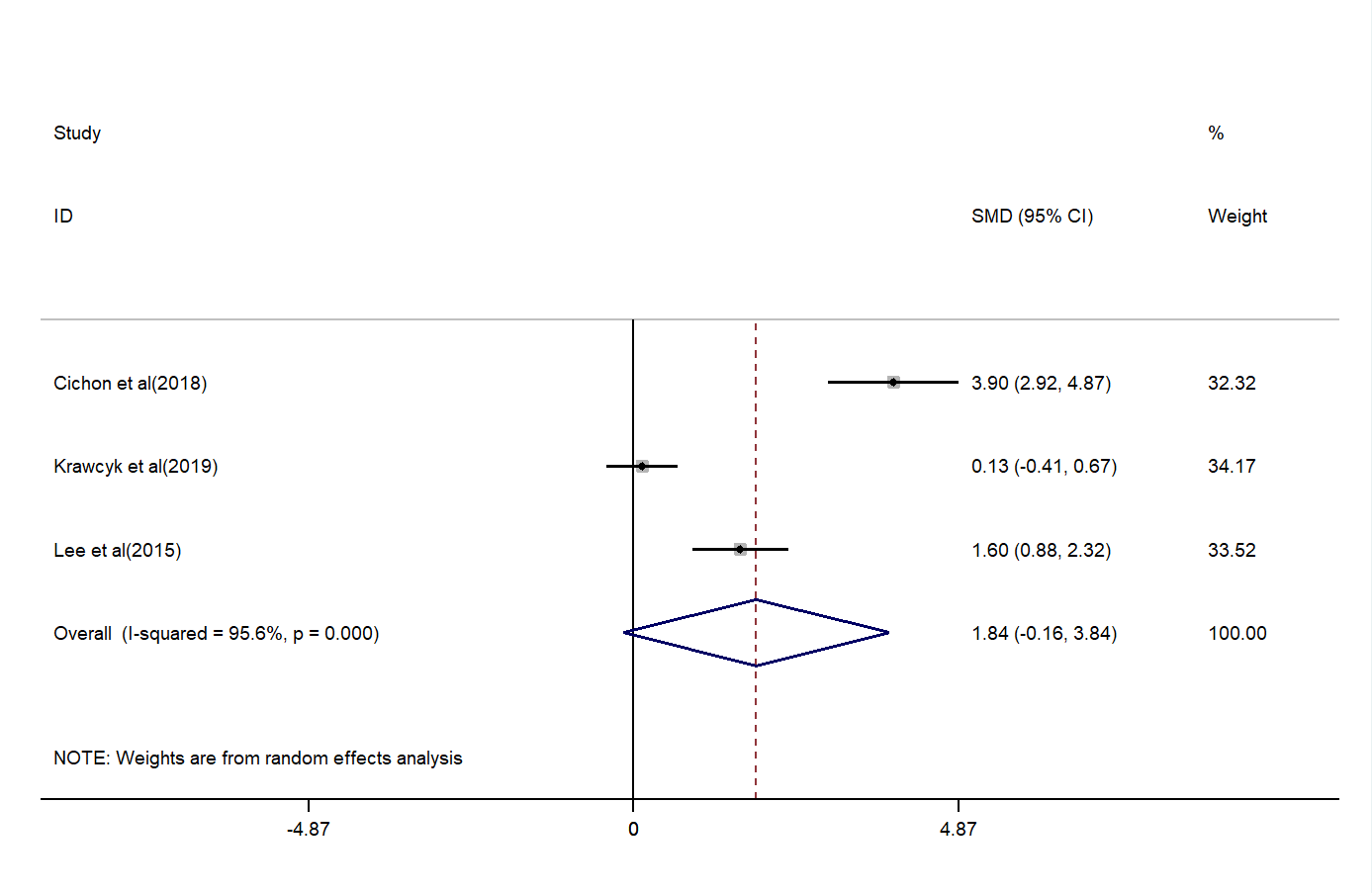


**Supplementary Figure 9** Forest plot of the effect of rehabilitation treatment on plasma vascular endothelial growth factor (VEGF) in stroke patients.


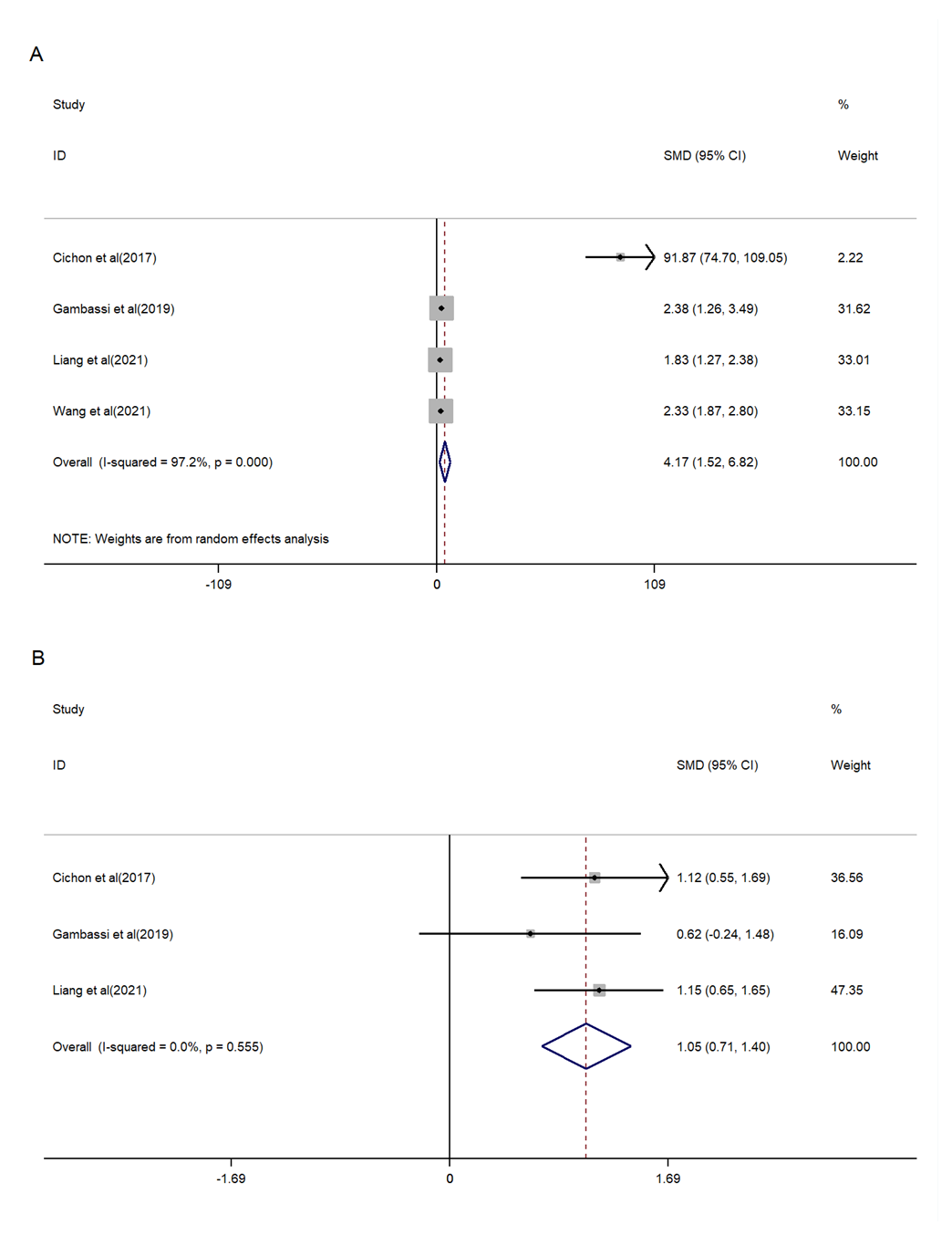


**Supplementary Figure 10** A. Forest plot of the effect of rehabilitation treatment on peripheral blood Superoxide Dismutase (SOD) in stroke patients; B. Forest plot of the effect of rehabilitation therapy on functional recovery in stroke patients in the peripheral blood SOD Study.


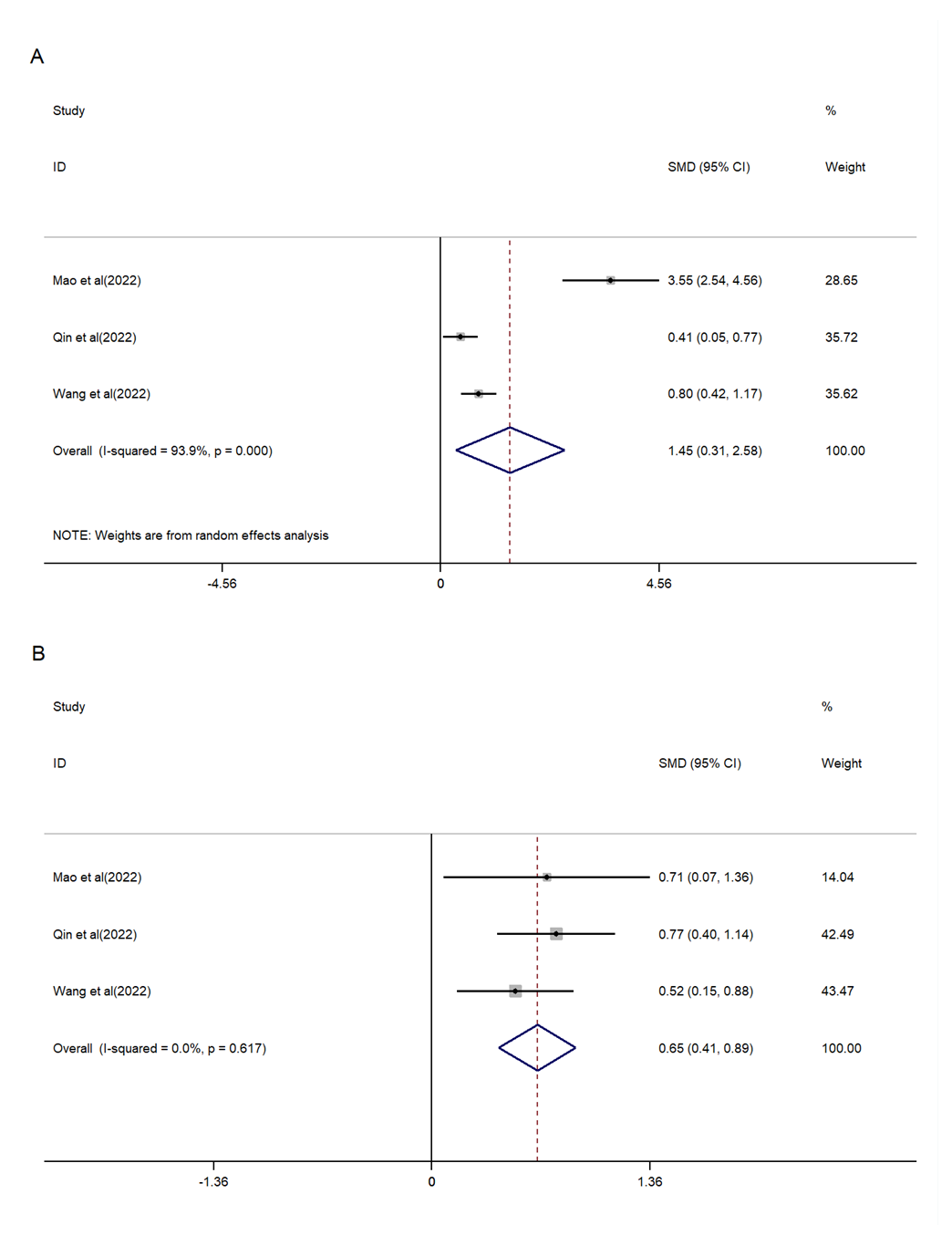


**Supplementary Figure 11** A. Forest plot of the effect of rehabilitation treatment on peripheral blood albumin (ALB) in stroke patients; B. Forest plot of the effect of rehabilitation therapy on functional recovery in stroke patients in the peripheral blood ALB Study.


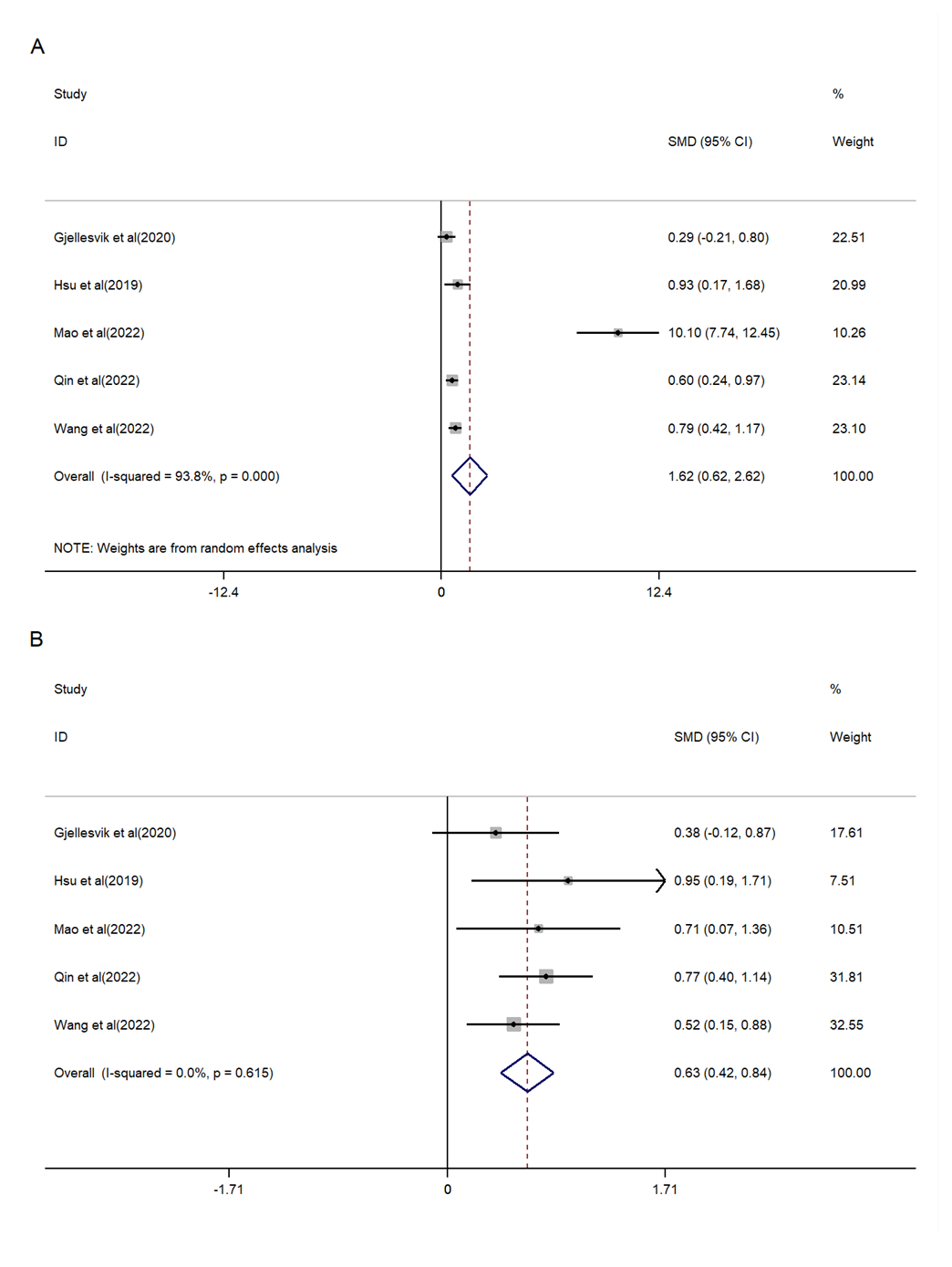


**Supplementary Figure 12** A. Forest plot of the effect of rehabilitation treatment on peripheral blood Hemoglobin (HB) in stroke patients; B. Forest plot of the effect of rehabilitation therapy on functional recovery in stroke patients in the peripheral blood HB Study.


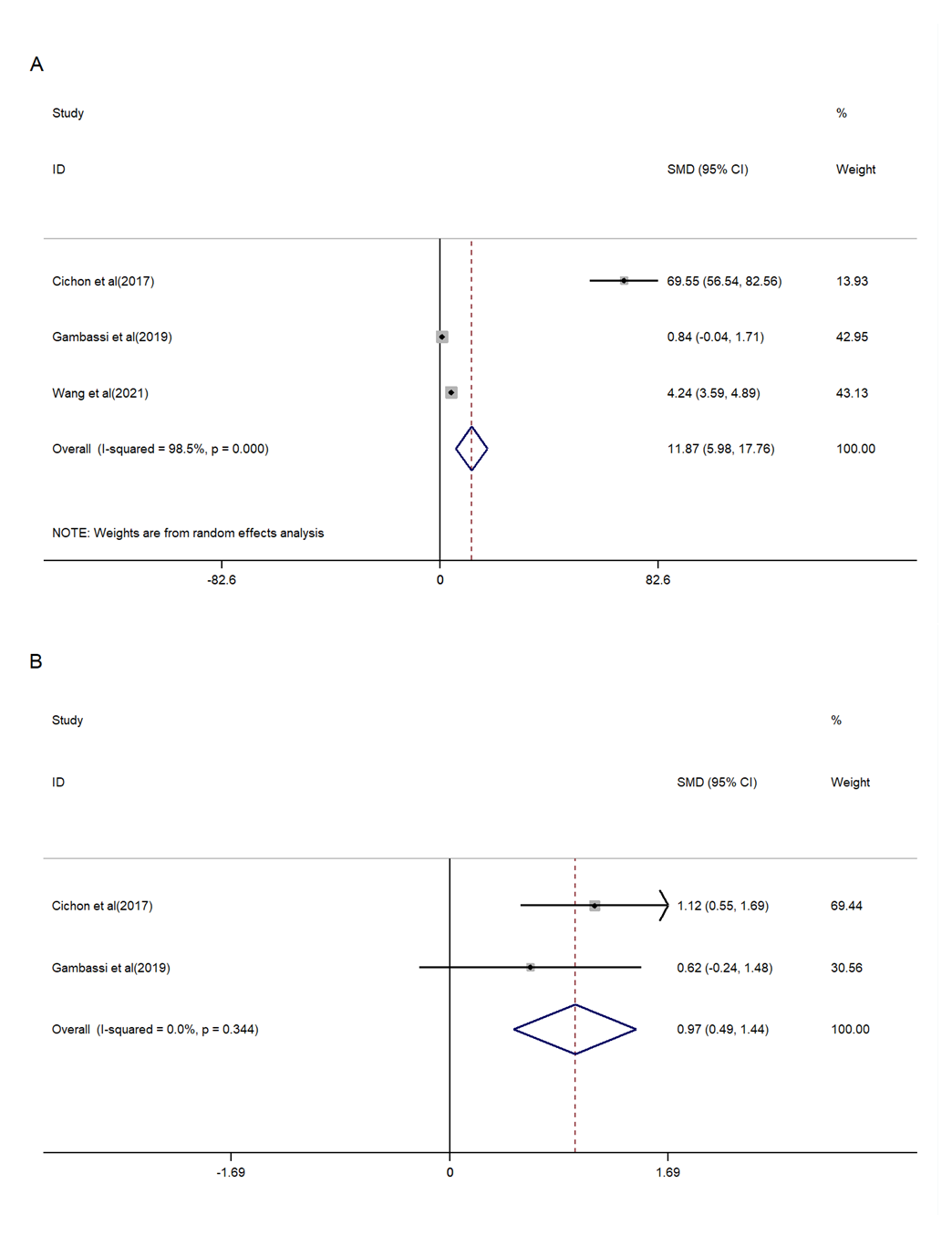


**Supplementary Figure 13** A. Forest plot of the effect of rehabilitation treatment on peripheral blood catalase (CAT) in stroke patients; B. Forest plot of the effect of rehabilitation therapy on functional recovery in stroke patients in the peripheral blood CAT Study.


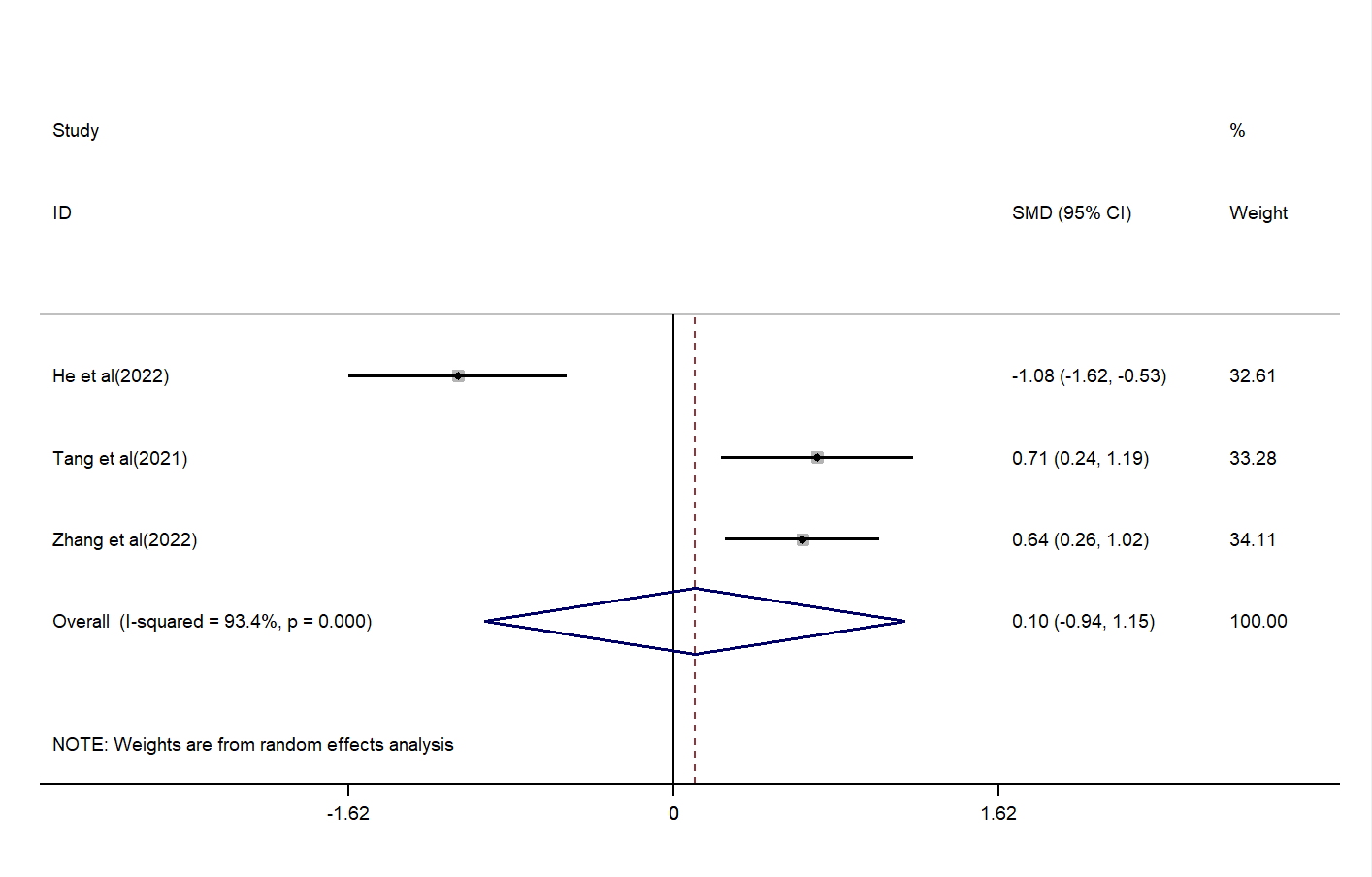


**Supplementary Figure 14** Forest plot of the effect of rehabilitation treatment on peripheral blood calcitonin-gene-related peptide (CGRP) in stroke patients.


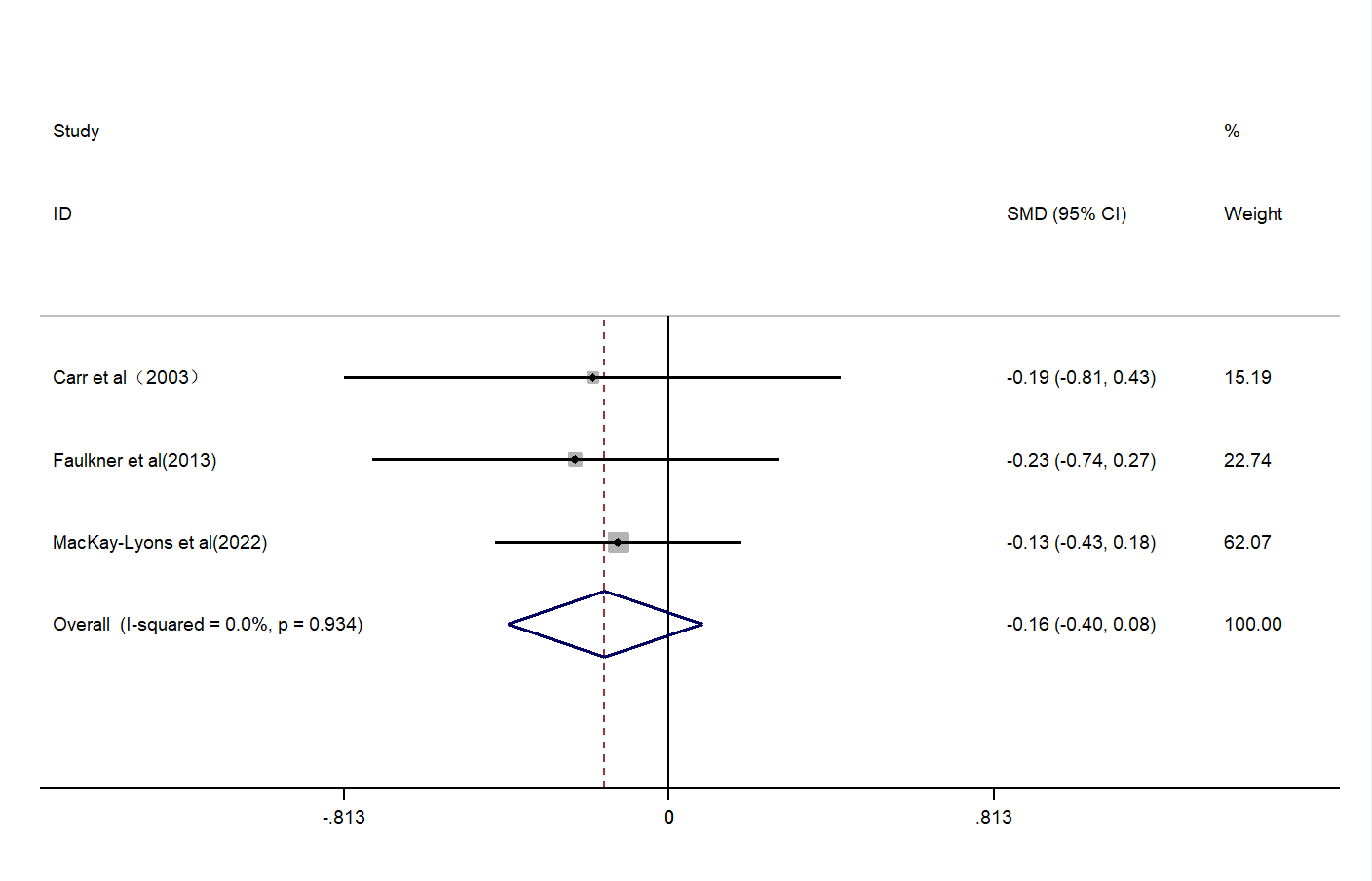


**Supplementary Figure 15** Forest plot of the effect of rehabilitation treatment on peripheral blood glucose (fasting) in stroke patients.


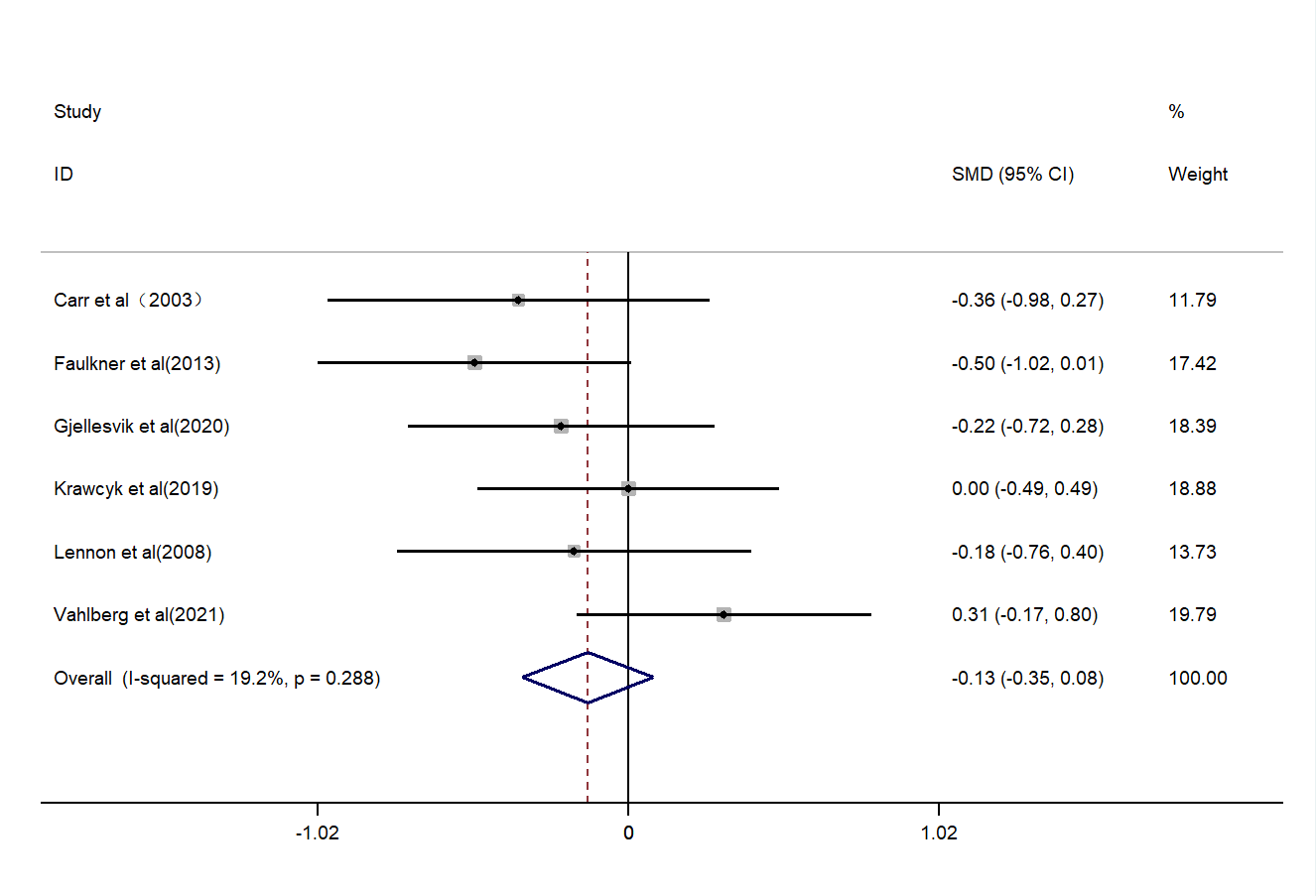


**Supplementary Figure 16** Forest plot of the effect of rehabilitation treatment on peripheral blood total cholesterol (TC) in stroke patients.


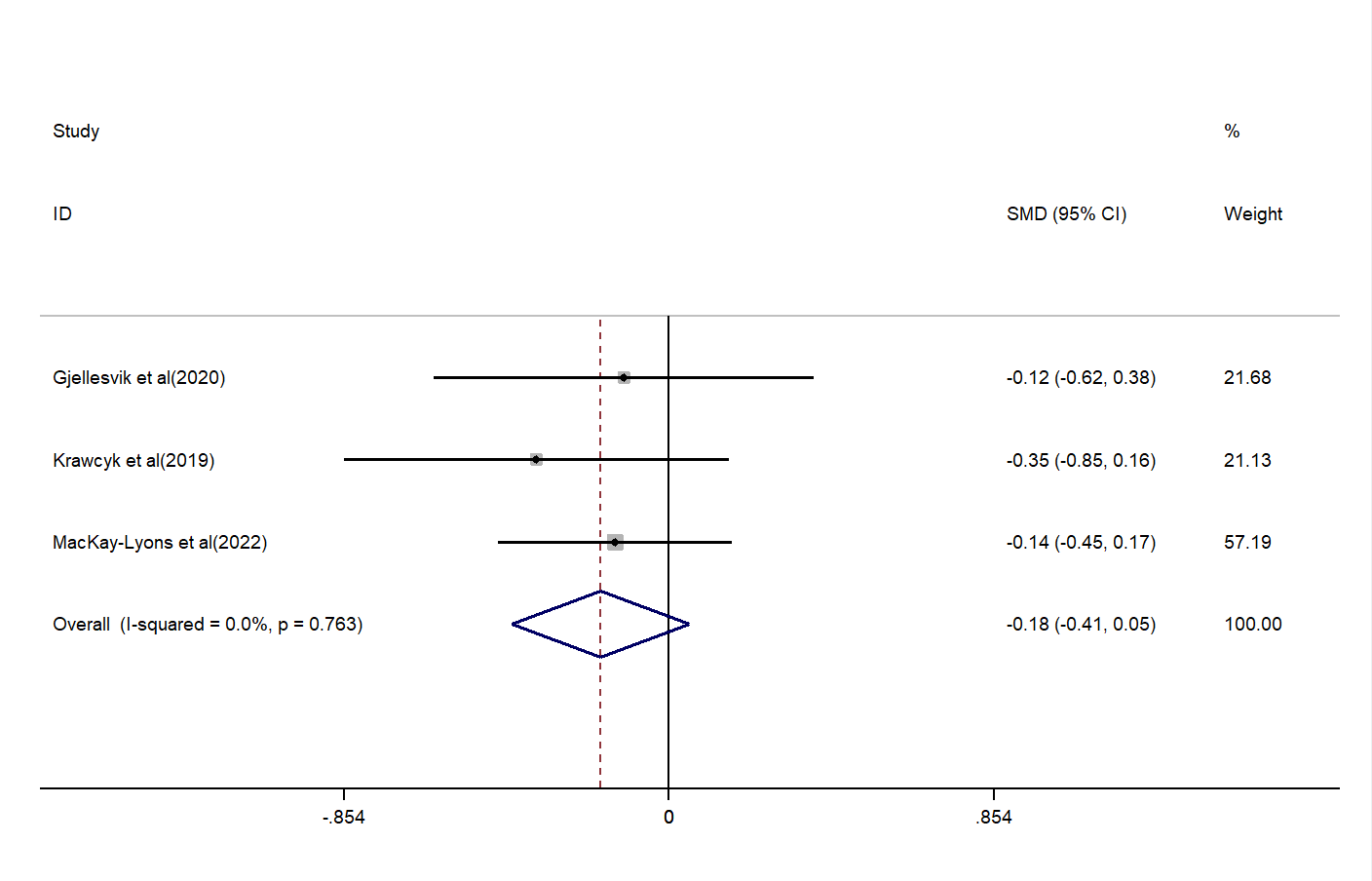


**Supplementary Figure 17** Forest plot of the effect of rehabilitation treatment on peripheral blood Triglyceride (TG) in stroke patients.


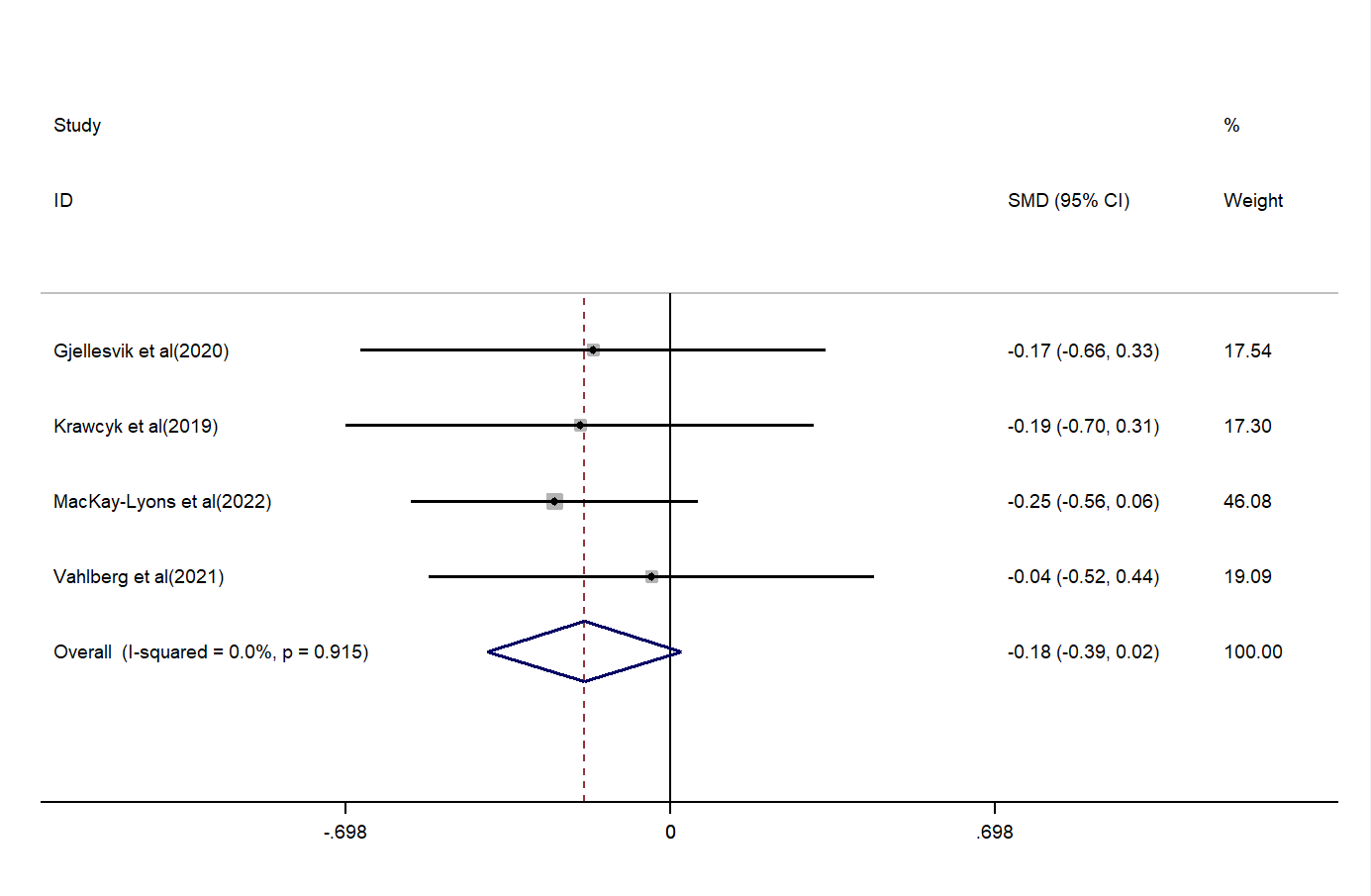


**Supplementary Figure 18** Forest plot of the effect of rehabilitation treatment on peripheral blood low density lipoprotein (LDL) in stroke patients.


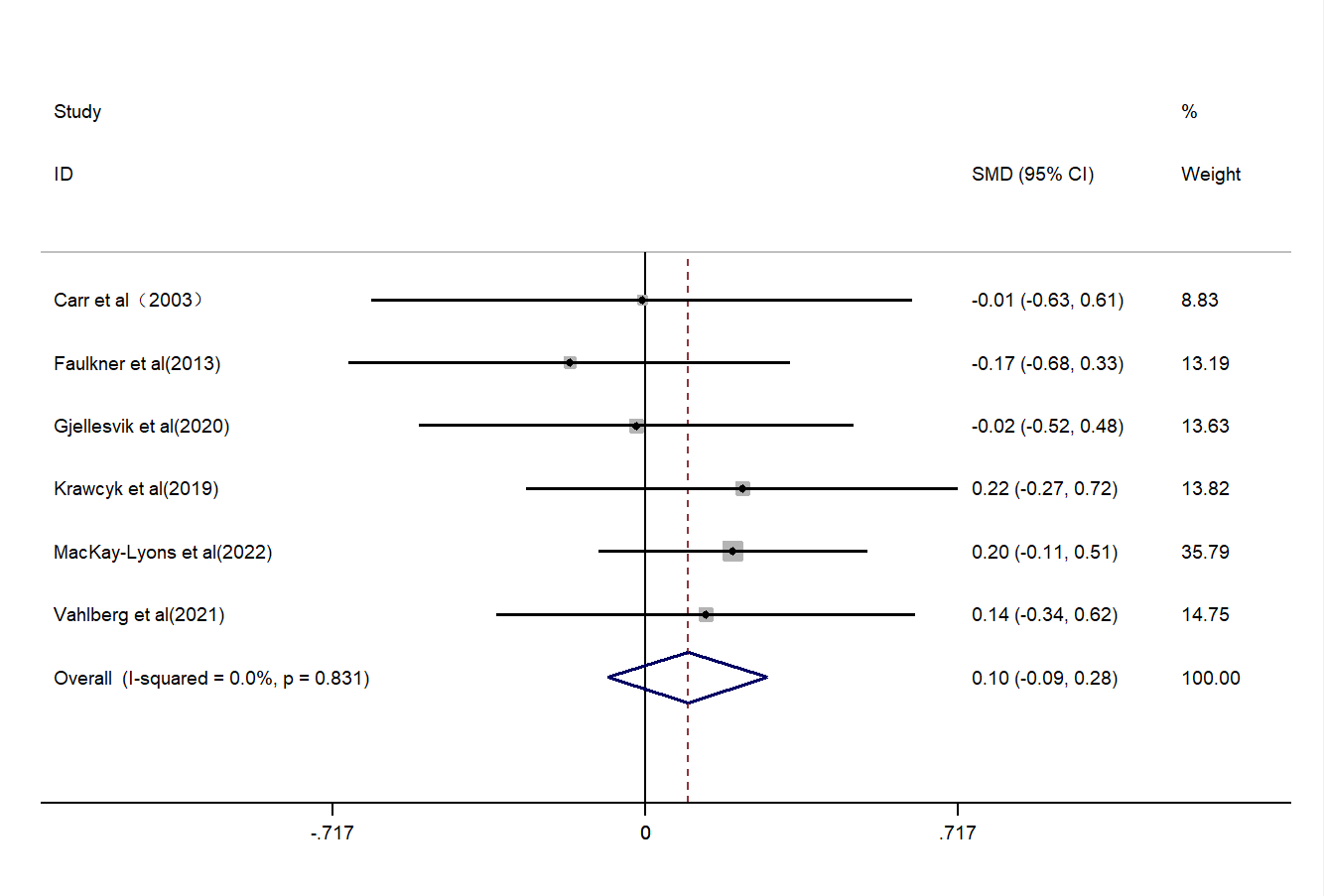


**Supplementary Figure 19** Forest plot of the effect of rehabilitation treatment on peripheral blood high density lipoprotein (HDL) in stroke patients.

**Reference**

1. Bai G, Jiang L, Huan S, Meng P, Wang Y, Pan X, et al. Study on Low-Frequency Repetitive Transcranial Magnetic Stimulation Improves Speech Function and Mechanism in Patients With Non-fluent Aphasia After Stroke. Frontiers in aging neuroscience. 2022;14:883542.doi:10.3389/fnagi.2022.883542

2. Bai G, Jiang L, Ma W, Meng P, Li J, Wang Y, et al. Effect of Low-Frequency rTMS and Intensive Speech Therapy Treatment on Patients With Nonfluent Aphasia After Stroke. The neurologist. 2021;26(1):6-9.doi:10.1097/nrl.0000000000000303

3. Bintang AK, Akbar M, Amran MY, Hammado N. The effect of high-and low-frequency repetitive transcranial magnetic stimulation therapy on serum brain-derived neurotropic factor level and motor ability in ischemic stroke patients: A single-center study. Open Access Macedonian Journal of Medical Sciences. 2020;8(B):198-204.doi:10.3889/OAMJMS.2020.3531

4. Carr M, Jones J. Physiological effects of exercise on stroke survivors. Topics in. 2003;9(4):57-64.doi:10.1310/0J2K-MDNX-1Q0L-8LX6

5. Cichon N, Bijak M, Czarny P, Miller E, Synowiec E, Sliwinski T, et al. Increase in Blood Levels of Growth Factors Involved in the Neuroplasticity Process by Using an Extremely Low Frequency Electromagnetic Field in Post-stroke Patients. Frontiers in aging neuroscience. 2018;10.doi:10.3389/fnagi.2018.00294

6. Cichon N, Bijak M, Miller E, Saluk J. Extremely Low Frequency Electromagnetic Field (ELF-EMF) Reduces Oxidative Stress and Improves Functional and Psychological Status in Ischemic Stroke Patients. Bioelectromagnetics. 2017;38(5):386-96.doi:10.1002/bem.22055

7. Faulkner J, Lambrick D, Woolley B, Stoner L, Wong L-k, McGonigal G. Effects of Early Exercise Engagement on Vascular Risk in Patients with Transient Ischemic Attack and Nondisabling Stroke. Journal of Stroke & Cerebrovascular Diseases. 2013;22(8):E388-E96.doi:10.1016/j.jstrokecerebrovasdis.2013.04.014

8. Gambassi BB, Coelho-Junior HJ, Paixão Dos Santos C, de Oliveira Gonçalves I, Mostarda CT, Marzetti E, et al. Dynamic Resistance Training Improves Cardiac Autonomic Modulation and Oxidative Stress Parameters in Chronic Stroke Survivors: A Randomized Controlled Trial. Oxidative medicine and cellular longevity. 2019;2019:5382843.doi:10.1155/2019/5382843

9. Gjellesvik TI, Becker F, Tjønna AE, Indredavik B, Nilsen H, Brurok B, et al. Effects of High-Intensity Interval Training After Stroke (the HIIT-Stroke Study): A Multicenter Randomized Controlled Trial. Archives of physical medicine and rehabilitation. 2020;101(6):939-47.doi:10.1016/j.apmr.2020.02.006

10. He L, Chen X, Zhang Y. Clinical Effect of Hufu Copper Scraping on Shoulder-Hand Syndrome after Stroke. Emergency medicine international. 2022;2022:9165141.doi:10.1155/2022/9165141

11. Hsu CC, Tsai HH, Fu TC, Wang JS. Exercise Training Enhances Platelet Mitochondrial Bioenergetics in Stroke Patients: A Randomized Controlled Trial. Journal of clinical medicine. 2019;8(12).doi:10.3390/jcm8122186

12. Huang SC, Hsu CC, Fu TC, Chen CPC, Liao MF, Hsu CY, et al. Stepper-Based Training Improves Monocyte-Platelet Aggregation and Thrombin Generation in Nonambulatory Hemiplegic Patients. Medicine and science in sports and exercise. 2022;54(5):821-9.doi:10.1249/mss.0000000000002846

13. Krawcyk RS, Vinther A, Petersen NC, Faber J, Iversen HK, Christensen T, et al. Effect of Home-Based High-Intensity Interval Training in Patients With Lacunar Stroke: A Randomized Controlled Trial. Frontiers in neurology. 2019;10.doi:10.3389/fneur.2019.00664

14. Lee S, Kim W, Park J, Jang HH, Lee SM, Woo JS, et al. Effects of electroacupuncture on endothelial function and circulating endothelial progenitor cells in patients with cerebral infarction. Clinical and experimental pharmacology & physiology. 2015;42(8):822-7.doi:10.1111/1440-1681.12413

15. Lennon O, Carey A, Gaffney N, Stephenson J, Blake C. A pilot randomized controlled trial to evaluate the benefit of the cardiac rehabilitation paradigm for the non-acute ischaemic stroke population. Clinical Rehabilitation. 2008;22(2):125-33.doi:10.1177/0269215507081580

16. Liang Y, Lin J, Wang H, Li S, Chen F, Chen L, et al. Evaluating the efficacy of vitalstim electrical stimulation combined with swallowing function training for treating dysphagia following an acute stroke. Clinics. 2021;76.doi:10.6061/clinics/2021/e3069

17. Liu W, Ding W. Study on the efficacy and mechanism of paroxetine hydrochloride combined with repetitive transcranial magnetic stimulation in the treatment of post-stroke depression. International Journal of Clinical and Experimental Medicine. 2020;13(10):7881-8

18. Lu H, Zhang T, Wen M, Sun L. Impact of repetitive transcranial magnetic stimulation on post-stroke dysmnesia and the role of BDNF Val66Met SNP. Medical science monitor : international medical journal of experimental and clinical research. 2015;21:761-8.doi:10.12659/msm.892337

19. MacKay-Lyons M, Gubitz G, Phillips S, Giacomantonio N, Firth W, Thompson K, et al. Program of Rehabilitative Exercise and Education to Avert Vascular Events After Non-Disabling Stroke or Transient Ischemic Attack (PREVENT Trial): A Randomized Controlled Trial. Neurorehabilitation and neural repair. 2022;36(2):119-30.doi:10.1177/15459683211060345

20. Mao H, Lyu Y, Li Y, Gan L, Ni J, Liu L, et al. Clinical study on swallowing function of brainstem stroke by tDCS. Neurological sciences : official journal of the Italian Neurological Society and of the Italian Society of Clinical Neurophysiology. 2022;43(1):477-84.doi:10.1007/s10072-021-05247-6

21. Qin L, Zhang XX, Jin X, Cui CH, Tang CZ. The effect of acupuncture on enteral nutrition and gastrointestinal dynamics in patients who have suffered a severe stroke. Current neurovascular research. 2022.doi:10.2174/1567202619666220822123023

22. Tang X-l, Pan H-y. Muscle regions of meridians warm needling method plus pricking Jing-Well points for blood-letting in the treatment of shoulder-hand syndrome after stroke. Journal of Acupuncture and Tuina Science. 2021;19(4):291-9.doi:10.1007/s11726-021-1260-x

23. Utomo A, Wulan SMM, Wardhani IL. Effect of short period simultaneous stimulation of transcranial direct current stimulation on occupational therapy to brain-derived neurotrophic factor serum in stroke patients. Systematic Reviews in Pharmacy. 2020;11(6):979-82.doi:10.31838/srp.2020.6.138

24. Vahlberg BM, Lundstrom E, Eriksson S, Holmback U, Cederholm T. Potential effects on cardiometabolic risk factors and body composition by short message service (SMS)-guided training after recent minor stroke or transient ischaemic attack: post hoc analyses of the STROKEWALK randomised controlled trial. BMJ open. 2021;11(10).doi:10.1136/bmjopen-2021-054851

25. Wang HY, Zhu CH, Liu DS, Wang Y, Zhang JB, Wang SP, et al. Rehabilitation training improves cognitive disorder after cerebrovascular accident by improving BDNF Bcl-2 and Bax expressions in regulating the JMK pathway. European review for medical and pharmacological sciences. 2021;25(10):3807-21.doi:10.26355/eurrev_202105_25949

26. Wang J, Chang E, Jiang Y. Effects of vitamin C stimulation on rehabilitation of dysphagia after stroke: a randomized trial. European journal of physical and rehabilitation medicine. 2022;58(4):558-64.doi:10.23736/s1973-9087.22.07337-3

27. Wang J, Gong D, Gong Y. CLINICAL EFFECT OF ELECTROACUPUNCTURE COMBINED WITH DRUGS ON ACUTE CEREBRAL INFARCTION AND ITS EFFECT ON SERUM TNF- Α HORIZONTAL IMPACT. Acta Medica Mediterranea. 2022;38(3):1973-80.doi:10.19193/0393-6384_2022_3_303

28. Wang J, Wang C, Wu X, Ma T, Guo X. Effect of Hyperbaric Oxygen Therapy on Sleep Quality, Drug Dosage, and Nerve Function in Patients with Sleep Disorders after Ischemic Cerebral Stroke. Emergency medicine international. 2022;2022.doi:10.1155/2022/8307865

29. Wang X, Peng Y, Zhou H, Du W, Wang J, Wang J, et al. The Effects of Enriched Rehabilitation on Cognitive Function and Serum Glutamate Levels Post-stroke. Frontiers in neurology. 2022;13:829090.doi:10.3389/fneur.2022.829090

30. Wang Y, Li F, He M-J, Chen S-J. The effects and mechanisms of transcranial ultrasound stimulation combined with cognitive rehabilitation on post-stroke cognitive impairment. Neurological Sciences. 2022;43(7):4315-21.doi:10.1007/s10072-022-05906-2

31. Wang Y, Yuan S, Ma X, Tian X. Effect of Rehabilitation in Combination with Hyperbaric Oxygen Treatment on the Secretion of Neurotrophic Factors and Oxidative Stress in Recovery Phase of Cerebral Infarction Patients. Indian Journal of Pharmaceutical Sciences. 2021;83:166-70.doi:10.36468/pharmaceutical-sciences.spl.233

32. Xiong J, Zhang Z, Ma Y, Li Z, Zhou F, Qiao N, et al. The effect of combined scalp acupuncture and cognitive training in patients with stroke on cognitive and motor functions. NeuroRehabilitation. 2020;46(1):75-82.doi:10.3233/nre-192942

33. Zhang H, Kang T, Li L, Zhang J. Electroacupuncture Reduces Hemiplegia Following Acute Middle Cerebral Artery Infarction with Alteration of Serum NSE, S-100B and Endothelin. Current Neurovascular Research. 2013;10(3):216-21.doi:10.2174/15672026113109990005

34. Zhang J, Wu Ye, Huang Y, Zhang S, Xu L, Huang X, et al. Effect of the Mendelsohn maneuver and swallowing training in patients with senile vascular dementia complicated with dysphagia. Journal of International Medical Research. 2021;49(5).doi:10.1177/03000605211013198

35. Zhang L, Jin M, He Q, Liu X, Hao Y, Chen W. Effects of Ditan Tongmai Decoction in combination with acupuncture on post-stroke recovery based on electroencephalogram. Ceska a Slovenska Neurologie a Neurochirurgie. 2022;85(4):306-11.doi:10.48095/cccsnn2022306

36. Zhao CG, Sun W, Ju F, Jiang S, Wang H, Sun XL, et al. Analgesic Effects of Navigated Repetitive Transcranial Magnetic Stimulation in Patients With Acute Central Poststroke Pain. Pain and therapy. 2021;10(2):1085-100.doi:10.1007/s40122-021-00261-0

37. Zhao C-G, Ju F, Sun W, Jiang S, Xi X, Wang H, et al. Effects of Training with a Brain-Computer Interface-Controlled Robot on Rehabilitation Outcome in Patients with Subacute Stroke: A Randomized Controlled Trial. Neurology and therapy. 2022;11(2):679-95.doi:10.1007/s40120-022-00333-z

38. Cichon N, Bijak M, Synowiec E, Miller E, Sliwinski T, Saluk-Bijak J. Modulation of antioxidant enzyme gene expression by extremely low frequency electromagnetic field in post-stroke patients. Scandinavian Journal of Clinical & Laboratory Investigation. 2018;78(7-8):626-31.doi:10.1080/00365513.2018.1542540

39. Cichon N, Czarny P, Bijak M, Miller E, Sliwinski T, Szemraj J, et al. Benign Effect of Extremely Low-Frequency Electromagnetic Field on Brain Plasticity Assessed by Nitric Oxide Metabolism during Poststroke Rehabilitation. Oxidative medicine and cellular longevity. 2017;2017.doi:10.1155/2017/2181942

40. Zhao R, Wang C, Wang Y. Changes in serum cellular adhesion molecule and matrix metalloproteinase-9 levels in patients with cerebral infarction following hyperbaric oxygen therapy A case and intergroup control study. Neural Regeneration Research. 2008;3(11):1245-8

41. Cichon N, Rzeznicka P, Bijak M, Miller E, Miller S, Saluk J. Extremely low frequency electromagnetic field reduces oxidative stress during the rehabilitation of post-acute stroke patients. Advances in Clinical and Experimental Medicine. 2018;27(9):1285-93.doi:10.17219/acem/73699

42. Cichon N, Saluk-Bijak J, Miller E, Sliwinski T, Synowiec E, Wigner P, et al. Evaluation of the effects of extremely low frequency electromagnetic field on the levels of some inflammatory cytokines in post-stroke patients. Journal of rehabilitation medicine. 2019;51(11):854-60.doi:10.2340/16501977-2623

43. Li S. Application of Rehabilitation Therapy in Pulmonary Infection of Stroke Patients. Indian Journal of Pharmaceutical Sciences. 2021;83:51-5.doi:10.36468/pharmaceutical-sciences.spl.211

44. Feng X, Huang L, Wang Z, Wang L, Du X, Wang Q, et al. Efficacy of remote limb ischemic conditioning on poststroke cognitive impairment. Journal of integrative neuroscience. 2019;18(4):377-85.doi:10.31083/j.jin.2019.04.1192

45. Ji Z, Yu L, Wu W, Fang Q. Remote ischemic postconditioning promotes collateral circulation and down-regulates TLR4/NF-kappa B signaling pathway in patients with acute ischemic stroke

International Journal of Clinical and Experimental Medicine. 2020;13(1):246-52

46. Zhen X, Zheng Y, Hong X, Chen Y, Gu P, Tang J, et al. Physiological ischemic Training Promotes Brain collateral Formation and improves Functions in Patients with acute cerebral infarction. Frontiers in neurology. 2016;7.doi:10.3389/fneur.2016.00235

47. Li H, Ma J, Zhang J, Shi W-Y, Mei H-N, Xing Y. Repetitive Transcranial Magnetic Stimulation (rTMS) Modulates Thyroid Hormones Level and Cognition in the Recovery Stage of Stroke Patients with Cognitive Dysfunction. Medical Science Monitor. 2021;27.doi:10.12659/msm.931914

48. Pang MY, Lau RW, Yip SP. The effects of whole-body vibration therapy on bone turnover, muscle strength, motor function, and spasticity in chronic stroke: a randomized controlled trial. European journal of physical and rehabilitation medicine. 2013;49(4):439-50

49. Zheng C, Lee Y, Hu B, Wu J. A randomized controlled trial comparing electroacupuncture with manual acupuncture for motor function recovery after ischemic stroke. European Journal of Integrative Medicine. 2018;22:76-80.doi:10.1016/j.eujim.2018.08.005

50. Zhang X, Yuan Y, Kuang P, Wu W, Zhang F, Liu J. Effects of electro-acupuncture on somatostatin and pancreatic polypeptide in ischemic cerebrovascular diseases. Journal of traditional Chinese medicine = Chung i tsa chih ying wen pan. 1999;19(1):54-8
